# Efficient Assessment and Optimisation of Medium Components Influencing Extracellular Xylanase Production by *Pediococcus pentosaceus* G4 Using Statistical Approaches

**DOI:** 10.3390/ijms26157219

**Published:** 2025-07-25

**Authors:** Noor Lutphy Ali, Hooi Ling Foo, Norhayati Ramli, Murni Halim, Karkaz M. Thalij

**Affiliations:** 1 Department of Bioprocess Technology, Faculty of Biotechnology and Biomolecular Sciences, Universiti Putra Malaysia, Serdang 43400 UPM, Selangor, Malaysia or noor.ali@cihanuniversity.edu.iq (N.L.A.); yatiramli@upm.edu.my (N.R.); murnihalim@upm.edu.my (M.H.); 2 Department of Medical Microbiology, College of Science, Cihan University-Erbil, Erbil 44001, Iraq; 3 Lactic Acid Bacteria Biota Technology Research Program, Research Laboratory of Probiotics and Cancer Therapeutics, UPM-MAKNA Cancer Research Laboratory (CANRES), Institute of Bioscience, Universiti Putra Malaysia, Serdang 43400 UPM, Selangor, Malaysia; 4 Department of Food Sciences, College of Agriculture, Tikrit University, Tikrit 34001, Iraq; kthalij@tu.edu.iq

**Keywords:** xylanase production, response surface methodology, *Pediococcus pentosaceus*, optimisation

## Abstract

Xylanase is an essential industrial enzyme for degrading plant biomass, pulp and paper, textiles, bio-scouring, food, animal feed, biorefinery, chemicals, and pharmaceutical industries. Despite its significant industrial importance, the extensive application of xylanase is hampered by high production costs and concerns regarding the safety of xylanase-producing microorganisms. The utilisation of renewable polymers for enzyme production is becoming a cost-effective alternative. Among the prospective candidates, non-pathogenic lactic acid bacteria (LAB) are promising for safe and eco-friendly applications. Our investigation revealed that *Pediococcus pentosaceus* G4, isolated from plant sources, is a notable producer of extracellular xylanase. Improving the production of extracellular xylanase is crucial for viable industrial applications. Therefore, the current study investigated the impact of various medium components and optimised the selected medium composition for extracellular xylanase production of *P. pentosaceus* G4 using Plackett–Burman Design (PBD) and Central Composite Design (CCD) statistical approaches. According to BPD analysis, 8 out of the 19 investigated factors (glucose, almond shell, peanut shell, walnut shell, malt extract, xylan, urea, and magnesium sulphate) demonstrated significant positive effects on extracellular xylanase production of *P. pentosaceus* G4. Among them, glucose, almond shells, peanut shells, urea, and magnesium sulphate were identified as the main medium components that significantly (*p* < 0.05) influenced the production of extracellular xylanase of *P. pentosaceus* G4. The optimal concentrations of glucose, almond shells, peanut shells, urea, and magnesium sulphate, as determined via CCD, were 26.87 g/L, 16 g/L, 30 g/L, 2.85 g/L, and 0.10 g/L, respectively. The optimised concentrations resulted in extracellular xylanase activity of 2.765 U/mg, which was similar to the predicted extracellular xylanase activity of 2.737 U/mg. The CCD-optimised medium yielded a 3.13-fold enhancement in specific extracellular xylanase activity and a 7.99-fold decrease in production costs compared to the commercial de Man, Rogosa and Sharpe medium, implying that the CCD-optimised medium is a cost-effective medium for extracellular xylanase production of *P. pentosaceus* G4. Moreover, this study demonstrated a positive correlation between extracellular xylanase production, growth, lactic acid production and the amount of sugar utilised, implying the multifaceted interactions of the physiological variables affecting extracellular xylanase production in *P. pentosaceus* G4. In conclusion, statistical methods are effective in rapidly assessing and optimising the medium composition to enhance extracellular xylanase production of *P. pentosaceus* G4. Furthermore, the findings of this study highlighted the potential of using LAB as a cost-effective producer of extracellular xylanase enzymes using optimised renewable polymers, offering insights into the future use of LAB in producing hemicellulolytic enzymes.

## 1. Introduction

Xylanases are vital industrial enzymes, contributing approximately 75% of the enzyme market [1]. They catalyse the hydrolysis of the D-xylosidic linkages in xylan [2]. Xylanolytic enzymes have gained significant attention in biotechnological and respective industrial applications [3]. Xylanase produced by various bacterial cultures has been utilised in the food and feed industries [4]. However, the exorbitant costs of fermentation medium and production approaches mediated by microorganisms, specifically bacteria-based, limit the applications of xylanolytic enzymes [3]. To address this challenge, various attempts have been made to utilise low-cost substrates derived from renewable agro-wastes or agricultural biopolymers, which would help reduce production costs, organic waste and pollution, making the production of the xylanolytic enzymes a more environmentally beneficial approach [5].

Nutshells, such as those from almonds [6], walnuts, pistachios, hazelnuts [7] and peanuts [8], are often wasted, yet contain valuable organic compounds. These nutshells primarily consist of lignin (15–20%), hemicelluloses (25–30%), and cellulose (40–50%) [9]. Utilising renewable agricultural biopolymers as low-cost substrates for xylanase production using potential microorganisms can significantly reduce production costs, especially in large-scale industrial settings [10,11]. Despite providing nutrients for microbial growth, these polymers also induce xylanase production [12]. However, a challenge in utilising lignocellulose is that many microorganisms cannot directly metabolise renewable agricultural biopolymers for the desired product formation [13]. The degradation of nutshells via xylanolytic bacteria is not extensively documented. Therefore, continued efforts to identify highly active and stable xylanase-producing microorganisms are essential to enhance yield and the desired xylanolytic enzyme characteristics [14].

Currently, xylanase production costs primarily depend on the fermentation method. A well-designed growth medium is essential for successful microbial fermentation, impacting the formation of desired products [15,16]. A cost-effective medium formulation is critical for ensuring the economic viability of the fermentation process [17]. It is crucial to optimise the medium composition to achieve high yields while minimising production costs [14]. To meet the industrial demand for large-scale xylanase production, novel and cost-effective bioprocesses, such as the optimisation of growth medium, are required [18].

Optimisation techniques for medium composition include the conventional one-factor-at-a-time (OFAT) approach and advanced statistical and mathematical methods [14,19]. However, the OFAT approach is often laborious and time-consuming. Moreover, the OFAT cannot analyse the interaction effects of multiple factors to determine the optimal conditions. To overcome the limitations of the OFAT, mathematical and statistical methods, such as Plackett–Burman Design (PBD) and Central Composite Design (CCD) of response surface methodology (RSM), are commonly employed to efficiently determine the impact of numerous factors to optimise the bioprocess approach [20]. The statistical tool of PBD offers cost-effective variable determination and selection [21]. Meanwhile, the CCD of the RSM further investigates and optimises positive impact factors determined via the PBD approach, and subsequently, the interactions of the optimised positive factors will be used for the development of a bioprocess model for the efficient production of desired bioproducts [22,23].

As for xylanase production, Coman and Bahrim [24] reported the increased xylanase synthesis of *Streptomyces* sp. P12-137 by using wheat bran as a substrate. Moreover, Bibra, Kunreddy, and Sani [25] observed improved xylanase production using *Geobacillus* sp. strain DUSELR13 and lignocellulosic biomass, specifically prairie cordgrass and maize stover. Furthermore, Thite, Nerurkar, and Baxi [26] explored the optimal concentrations of agro-waste for xylanase production using *Bacillus safensis* M35 and *Bacillus altitudinis* J208. However, pathogenic microorganisms are a significant concern, driving the continued effort in the search for safer enzyme producers. Assessing the safety of enzyme-producing microorganisms is crucial to ensure that xylanase is produced by a non-pathogenic, non-toxic, and eco-friendly microorganism classified as Generally Recognised as Safe (GRAS), which is essential [27] for fulfilling the industrial demands of xylanase applications [14,28].

Lactic acid bacteria (LAB) have been known generally as GRAS microorganisms, noted for their safety profile and ability to produce a range of extracellular cellulolytic and hemicellulolytic enzymes, including protease, cellulase, xylanase, and mananase [29,30]. The impact of various renewable agro-waste biopolymers as growth medium components on xylanase production by LAB has not been extensively explored. However, Lee et al. [29] investigated the biotransformation of palm kernel cake biomass using LAB. Furthermore, Zabidi et al. [30] demonstrated the capability of various LAB strains isolated from Malaysian foods to biotransform lignocellulosic biomass by producing extracellular hemicellulolytic enzymes. Nevertheless, the potential of nutshells as a cost-efficient carbon source for xylanase production has not been documented. Thus, this study aimed to evaluate the effects of various nutshells and other medium components on growth and extracellular xylanase production by *Pediococcus pentosaceus* G4 isolated from the gundelia (*Gundelia tournefortii*) plant, using the PBD statistical approach. CCD statistical methodologies were subsequently employed to optimise positive medium components and to develop a bioprocess model to enhance extracellular xylanase production of *P. pentosaceus* G4.

## 2. Results and Discussion

### 2.1. Assessment of Medium Components by Plackett–Burman Design

The nutritional requirements of *P. pentosaceus* G4 for extracellular xylanase production were investigated using PBD. Each nutrient variable of PBD was assigned a value of +1 and −1, representing the lowest and highest values of each nutrient variable. Table 1 shows the specific extracellular xylanase activity of *P. pentosaceus* G4 that corresponds to the experimental run of PBD.

Overall, most experimental runs produced extracellular xylanase, except for runs 12, 18, and 19, indicating that the medium composition substantially affected extracellular xylanase production by the producer strain. In comparison, the medium composition of experimental run 16 exhibited the highest specific extracellular xylanase activity of 1.0329 U/mg, followed by the medium composition of experimental run 15, with specific extracellular xylanase activity of 0.6453 U/mg. Furthermore, the lowest specific extracellular enzyme activity was noted for the medium composition of experimental run 6, with an activity of 0.0121 U/mg. Overall, the extracellular xylanase activity produced by *P. pentosaceus* G4 using the medium composition of experimental run 16 (1.0329 U/mg) was significantly higher (*p* < 0.05) compared to the control MRS medium (0.8821 U/mg). Nonetheless, it is imperative to optimise the medium composition of experimental run 16 to further enhance the production of extracellular xylanase by *P. pentosaceus* G4.

Analysis of variance (ANOVA) (Table 2) was conducted to assess the adequacy of the model and the significance of each medium constituent for the production of extracellular xylanase by *P. pentosaceus* G4. The obtained *p*-value of the model (<0.0001) indicates that the model was highly significant (*p* < 0.01), suggesting a very low probability (0.01%) that the F-value of the model was due to noise. Furthermore, the value of the coefficient of determination, R^2^, of the model was 0.9983, implying that the model’s explanatory power could account for 99% of the variation in response. Moreover, the difference between the predicted R^2^ (0.9584) and the adjusted R^2^ (0.9921) was less than 0.2, suggesting that the model demonstrated a high degree of fitness. According to Li et al. [31], a higher R^2^ value indicates a stronger correlation between the experimental and predicted values. In addition, it is worth noting that the precision value of the current model (48.2251) exceeded the threshold of 4, indicating that the model has sufficient accuracy to be effectively used for navigating the design further.

The ANOVA of extracellular xylanase production (Table 2) showed that glucose, almond shell, peanut shell, hazelnut shell, pistachio shell, walnut shell, malt extract, xylan, peptone, yeast extract, meat extract, urea, sodium acetate, magnesium sulphate and dipotassium hydrogen phosphate contribute significantly (*p* < 0.05) to extracellular xylanase production of *P. pentosaceus* G4. The regression model of the medium constituent effects on the specific extracellular xylanase activity (Y) of *P. pentosaceus* G4 can be represented using coded symbols (A–S) according to regression Equation (1) as follows:

(1) $$\begin{array}{l} Y=0.2051+0.1030A+0.0976B+0.0967C-0.0255D-0.0607E+0.0315F+\\ 0.0164G+0.0975H-0.0441J-0.1054K-0.0431L+0.0832N-0.0380P+\\ 0.0364Q-0.0245S \end{array}$$

**Figure 1:**
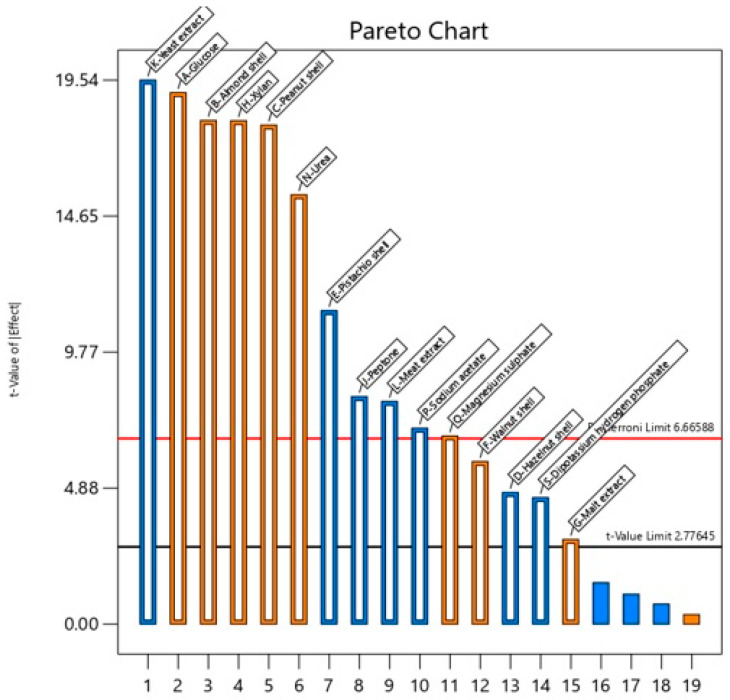
Pareto chart of the effects of different medium components on extracellular xylanase production using *P. pentosaceus* G4. The absolute t-values of the Pareto chart represent the effect levels of each growth medium. The orange bar indicates positive effects, and the blue bar indicates negative effects.

A Pareto chart of Figure 1 was subsequently created to determine the scale and significance of each growth medium investigated in the PBD for xylanase production by *P. pentosaceus* G4. The absolute t-values of the Pareto chart represent the effect levels of each growth medium, with the orange bar indicating positive effects and the blue bar indicating negative effects. The significance levels of four factors, including potassium nitrate, ammonium citrate, manganese sulphate and Tween 80, were noted as being below the significance threshold, with low t-values at the far right of Figure 1, indicating that they did not have a positive impact on extracellular xylanase production by *P. pentosaceus* G4. Therefore, these factors were not selected from the subsequent optimisation study.

Pistachio shell, peptone, yeast extract, meat extract, sodium acetate and dipotassium hydrogen phosphate demonstrated a suppressive impact, but hazelnut shell did not. The other growth medium components exhibited a positive influence on extracellular xylanase production. Among the eight positive effects of the growth medium, seven showed significant effects with *p*-values below 0.01, except for malt extract, which displayed significance at a *p*-value of less than 0.05.

The examined carbon sources, including glucose, almond shell, peanut shell, walnut shell and xylan, induced extracellular xylanase production, with glucose exhibiting the most significant impact, as shown in Figure 1. The substantial effects of diverse carbon sources on extracellular xylanase production indicated that the carbon source was essential for enzyme production by *P. pentosaceus* G4. Moreover, *P. pentosaceus* G4 demonstrated the capability to utilise various carbon sources for its extracellular xylanase production. Likewise, the nitrogen sources utilised in this investigation, particularly urea, demonstrated the most significant positive effect (*p* < 0.01), followed by malt extract, which also significantly enhanced extracellular xylanase production of *P. pentosaceus* G4. The considerable impact of nitrogen sources on extracellular xylanase production suggested that *P. pentosaceus* G4 has fastidious nutritional demands. The minerals also markedly improved the extracellular xylanase production of *P. pentosaceus* G4. Among the minerals, magnesium sulphate demonstrated significance at a *p*-value of less than 0.01. In contrast, sodium acetate and dipotassium hydrogen phosphate displayed significant adverse effects on the extracellular xylanase production of *P. pentosaceus* G4.

It is worth noting that among the eight factors that exhibited a positive impact on extracellular xylanase production, five of them, including glucose, almond shell, peanut shell, urea and magnesium sulphate, demonstrated high coefficient values. Hence, they were selected for the subsequent optimisation study mediated via the CCD of RSM.

Carbon sources have been reported to be of utmost importance in the production of xylanase [20,32,33,34,35] as a fundamental constituent of the medium for cellular and metabolic processes. The type of carbon source significantly affects enzyme production by providing energy and inducing substances [32,33,36,37]. This study demonstrated that glucose, xylan, almond shell, peanut shell and walnut shell have a positive effect on extracellular xylanase production. Conversely, hazelnut shells and pistachio shells showed a negative impact on extracellular xylanase production.

Among the carbon sources, it was noted that the xylanase production of *P. pentosaceus* G4 was positively affected when xylan was used as an inducer in the production medium. Similarly, Bedade et al. [32] reported the highest xylanase activity of 13.09 U/mL from *Tuber maculatum* mycelium using xylan as the sole carbon source, as well as by many other microorganisms, including *Bacillus cereus* BSA-1, *Bacillus thermantarcticus* [38], *Micrococcus* sp. SAMRC-UFH3 [39], *Bacillus pumilus* [40] and *Geobacillus* sp. strain WSUCF1 [41]. Higher hydrolytic conversion of birchwood xylan was reported for *Bacillus* sp. BP-23 [42], *Bacillus firmus* [43], *Gracilibacillus* sp. TSCPVG [44], *Gracilibacillus* sp. TSCPVG [45], *Sporotrichum thermophil* [12], *Jonesia denitrificans* BN-13 [46], *Rhodothermus marinus* IT1376 [47], *Kluyvera* sp. OM3 [48] and *Pseudomonas mohnii* [49]. However, because xylan is a complex polymer, the enzyme production required to hydrolyse such a complex structure may increase only after the growth of the microorganisms reaches a certain level [50].

However, it is imperative to decrease the enzyme production time. Hence, one of the strategies to reduce the xylanase enzyme production time is to add glucose and xylan to the production medium, where glucose can be easily hydrolysed for growth to produce biomass to synthesise the xylanase enzyme rapidly [50]. In the current study, glucose has the most significant positive impact on the extracellular xylanase synthesis by *P. pentosaceus* G4. The results of the present study are in agreement with Pasalari and Homaei [51], who reported the production of extracellular xylanase by *Bacillus subtilis* HR05 using glucose as the sole carbon source. The utilisation of glucose as a carbon source has been demonstrated to promote bacterial growth effectively and significantly augment the production of xylanase [13,50,52,53]. The LAB use glucose as their primary carbon source, enabling varied metabolic activities that aid cell growth, in which their glycolysis metabolic pathway begins with glucose, producing metabolic byproducts [54]. However, the results of the present investigation concerning the positive effect of glucose contrast with the findings of Mendonça et al. [55], who found that glucose reduced the xylanase yields of *Escherichia coli* due to catabolite suppression, accompanied by the production of acetic acid [56], which could be a potential inhibitory effect of quickly metabolisable substrates on enzyme production [50].

Interestingly, the other two carbon sources (almond shells and peanut shells), which positively affected extracellular xylanase production, were renewable biopolymers. Singh et al. [57] expressed endoxylanase in *Aspergillus oryzae* to obtain low polymerisation xylooligosaccharides from almond shells containing around 27.8% xylan [58,59]. Hence, almond shells can be employed as an alternative carbon source to induce xylanase synthesis, attributed to their significant xylan content. Furthermore, the positive effect of peanut shells on extracellular xylanase productivity of *P. pentosaceus* G4 was in agreement with the findings of Cho, Hatsu, and Takamizawa [60], who performed the enzymatic hydrolysis of peanut shells using xylanase from *Penicillium* sp. and *Rhizomucor pusillus* to obtain D-xylose. Similarly, the stimulatory effect of peanut shells on the production of lignocellulolytic enzymes has been reported for *Talaromyces amestolkiae* [61] and *Aspergillus awamori* [62,63]. According to Raju, Kumarappa, and Gaitonde [64], peanut shells contain approximately 18.7% hemicellulose, glucose, and approximately 3.5% xylan [60,63,65], which has been proven to be a valuable carbon source for xylanase synthesis.

The cost of large-scale industrial enzyme production is primarily related to the cost of substrates. Therefore, using renewable agricultural biopolymers as substrates is an integral approach in reducing production costs for industrial enzymes [66]. To date, various agro residues have been tested as alternative carbon sources for xylanase production [36]. Nonetheless, the utilisation of economical nutshells as an alternative carbon source for extracellular xylanase production by LAB has not been documented previously. The present study reveals, for the first time, that *P. pentosaceus* G4 could utilise these underutilised renewable biomass polymer resources for extracellular xylanase production. This not only presents a novel substrate source but also enhances the valorisation of inexpensive agro-industrial byproducts, potentially reducing enzyme manufacturing costs and boosting sustainable bioprocessing.

The findings of our experiment indicated that among all the nitrogen sources, the highest extracellular xylanase activity was achieved using urea and malt extract, which demonstrated a positive effect on extracellular xylanase production. In contrast, peptone, yeast extract, and meat extract had a negative impact on extracellular xylanase production. This is consistent with the findings of Paul, Nayak, and Thatoi [67], who demonstrated that nitrogen sources greatly influence xylanase production, in that *Pseudomonas mohnii* exhibited a maximum xylanase activity of 21.72 IU/mL when the growth medium contained 0.4% urea. Seyis and Aksoz [50] also obtained comparable findings, suggesting that the inclusion of urea as an additional nitrogen source resulted in a slight increase in xylanase activity, elevating it from 711.5 U/mg to 760.0 U/mg, indicating that urea is essential when the primary goal is to maximise xylanase production. Similar results were also reported by Kumar et al. [68], who demonstrated that urea at a concentration of 1.2 M induced a change in the secondary and tertiary structure of xylanase, potentially increasing its flexibility, particularly in the active region of the xylanase enzyme. This structural alteration ultimately leads to an increase in enzyme activity. Moreover, the production of xylanase by *Kluyveromyces lactis* was boosted in the presence of urea [69]. These findings are in contrast with the study conducted by Sá-Pereira et al. [70], who reported that the utilisation of urea as a nitrogen source led to the significant inhibition of xylanase production of *Bacillus subtilis*.

Urea could have a beneficial impact on xylanase production by providing the nitrogen required for growth and metabolic activities, including the synthesis of amino acids and nucleotide bases. However, urea might influence the pH of the fermentation medium, which in turn affects the production of the enzyme or leads to the accumulation of ammonia [3,36]. Hence, the most suitable concentration of urea may vary depending on the particular producer microorganisms employed for xylanase production. Therefore, it is essential to further optimise the concentration of urea for xylanase production.

According to Marimuthu, Sorimuthu, and Muruganantham [71], the most significant production of xylanase by *Bacillus subtilis* was attained when employing a 3% malt extract as the nitrogen source. Ellatif et al. [72] demonstrated that enzyme production using *Trichoderma harzianum* was enhanced with the inclusion of malt extract, indicating that malt extract promotes the highest level of enzyme activity as an enzyme inducer. In contrast, our findings did not reveal the stimulating effect of malt extract on xylanase production by *P. pentosaceus* G4. Similarly, Battan, Sharma, and Kuhad [73] reported that malt extract suppressed the production of xylanase of *Bacillus pumilus* ASH. Additionally, Sanghi et al. [74] also found that the use of malt extract contributed to a decrease in xylanase activity in *Bacillus subtilis* ASH.

On the other hand, peptone had an adverse effect on extracellular xylanase production by *P. pentosaceus* G4. This finding is consistent with Adhyaru, Bhatt, and Modi [75], who reported that the peptone did not show a stimulatory effect on xylanase production by *Bacillus altitudinis* DHN8. Our results contrast with those of Palaniswamy et al. [76] and Battan et al. [73], who reported that peptone enhanced xylanase production of *Bacillus pumilus* ASH, *Penicillium fellutanum*, and *Acremonium furcatum*.

Peptone is a water-soluble protein, a heterogeneous mixture of peptides with a small amount of free amino acids [77,78]. It has the potential to contain suppressive components that inhibit the synthesis of xylanase, attributed to their interference with bacterial growth and metabolism. Additionally, the high concentration of nitrogen in the growth medium has the potential to disrupt the equilibrium of enzyme production. Hence, it may potentially compete with essential nutrients, limiting the availability of the crucial elements required for xylanase production. The combined influence of these variables contributes to the adverse impact of peptone on xylanase synthesis.

The remaining two nitrogen sources, yeast extract and meat extract, also exhibited a negative impact on xylanase production of *P. pentosaceus* G4. Sá-Pereira et al. [70] reported similar observations in their experiments. They found that xylanase production of *Bacillus subtilis* was low when yeast extract was used in the production medium. However, yeast extract has been reported to increase xylanase production of *Bacillus mojavensis*. Moreover, the combined effects of yeast extract and beef extract in the production medium enhanced xylanase activity of this bacterium, reaching 213.218 IU/mL [79]. Likewise, beef extract improved xylanase production of *Bacillus circulans* D1 [80]. The impact of these extracts on xylanase synthesis may vary depending on the specific bacterial producer. The current study indicates that among all the evaluated nitrogen sources, urea demonstrated the most significant positive coefficient in extracellular xylanase production of *P. pentosaceus* G4. This reveals the significant stimulatory impact of urea on enzyme production. Although urea is generally linked to the alkalinisation of the medium, which is often detrimental to acidophilic bacteria, the studied *P. pentosaceus* G4 exhibited exceptional tolerance. This highlights a potential metabolic adaptation that enables efficient nitrogen uptake for bacterial growth and enzyme production.

The mineral sources involved in this study were magnesium sulphate, sodium acetate and dipotassium hydrogen phosphate. The analysis of the results showed that only magnesium sulphate had a significant positive effect on the extracellular xylanase production of *P. pentosaceus* G4. In contrast, sodium acetate and dipotassium hydrogen phosphate exhibited a negative impact on xylanase enzyme production. Atalla et al. [81] observed a considerable increase in xylanase activity when the MgSO_4_ concentration was 2.5 g/L. Additionally, Ravindran, Williams, and Jaiswal [82] demonstrated that the inclusion of 0.03 g of MgSO_4_ resulted in the enhancement of xylanase synthesis of *Aspergillus niger*. Similarly, Geetha and Gunasekaran [83] demonstrated that MgSO_4_·7H_2_O also had a significant positive effect on xylanase production of *Bacillus pumilus* B20. Long et al. [84] successfully determined that the optimal concentration of 0.08% MgSO_4_ resulted in a significant increase in xylanase activity of *Trichoderma orientalis*, reaching a maximum of 269.4 IU/mL. Nevertheless, Senthilkumar et al. [85] concluded that the addition of MgSO_4_ did not provide a statistically significant effect on the production of xylanase of *Aspergillus fischeri*.

Magnesium sulphate plays several roles in the synthesis of xylanase. Firstly, magnesium is a crucial cofactor in several enzyme operations, thus serving diverse intracellular physiological functions [86]. The metal ion cofactor is known for its tendency to form stable complexes with phosphate-containing species, including ATP, in physiological conditions [87]. Moreover, Mg^2+^ has a positive effect on the stability of ribosomes and cellular membranes, hence leading to an enhancement in xylanase activity [81]. The presence of magnesium ions is crucial in stabilising enzyme structures and facilitating their catalytic activities. Similarly, a higher magnesium flux has been linked to the promotion of bacterial growth and their survival [88]. The importance of magnesium ions in maintaining barrier permeability and retaining the integrity of the cell membrane and various cellular structures of bacterial cells has been highlighted by Lusk, Williams, and Kennedy [89].

In the current study, sodium acetate was found to have an adverse effect on the extracellular xylanase production of *P. pentosaceus* G4. Microbial cells are affected adversely by the released acetate anions, which impede their growth by raising the inner turgor pressure [90]. The stress may alter the physiology and metabolism of the cell. The impact of sodium acetate on xylanase production is highly variable and influenced by factors such as the concentration of sodium acetate and its interaction with other components in the growth medium. These interactions can either enhance or inhibit overall microbial growth, subsequently affecting xylanase production. To achieve the maximum level of xylanase production while mitigating any potential adverse effects, it is essential to optimise the sodium acetate according to the purpose of the fermentation process.

The addition of K_2_HPO_4_ to the production media had an adverse effect on the synthesis of extracellular xylanase production of *P. pentosaceus* G4. This finding was consistent with the results of Geetha and Gunasekaran [83], who also reported the adverse effects of K_2_HPO_4_ on xylanase production of *Bacillus pumilus* B20. The presence of phosphate ions has the potential to increase the pH of the growth medium, which might inhibit the growth of acidophilic bacteria, including LAB [91]. Subsequently, this could potentially adversely affect the ability of *P. pentosaceus* G4 to synthesise various enzymes. Our findings indicated that magnesium sulphate significantly enhanced extracellular xylanase production of *P. pentosaceus* G4, whereas other minerals demonstrated inhibitory effects. The discrepancies in the effects of minerals suggest that the impact of metal ions is likely dependent on the specific enzyme and producer microorganisms, which require mineral cofactors that promote cellular metabolism, including enzyme stability and activity.

### 2.2. Optimisation of Selected Medium Compositions by Central Composite Design

The concentrations of glucose, almond shell, peanut shell, urea, and magnesium sulphate, which have significant effects on extracellular xylanase production of *P. pentosaceus* G4, were subsequently optimised by employing the CCD of RSM. The CCD examined the impact of the five selected medium compositions on four responses: specific extracellular xylanase activity, cell population, lactic acid concentration and utilised sugar. Additionally, the initial and final pH were measured for each experimental run of CCD to evaluate the effect of pH on the four selected responses. The concentrations of glucose, almond shell, peanut shell, urea, and magnesium sulphate were designated as high (+1), low (−1), central (0) and two axial points (±α). The CCD suggested 50 experimental runs, as shown in Table 3.

#### 2.2.1. Extracellular Xylanase Production of *P. pentosaceus* G4

In general, the specific extracellular xylanase activity was significantly (*p* < 0.05) highest in experimental run 22 (2.9243 U/mg), followed by experimental run 42 (2.7889 U/mg), which consisted of glucose, almond shell, peanut shell, and urea. In comparison, the extracellular xylanase activity of the control MRS medium was 0.8809 U/mg. The data from the CCD experimental runs were subsequently analysed to determine the optimal model for describing the relationship between glucose, almond shell, peanut shell, urea, and magnesium sulphate on the specific extracellular xylanase activity, cell population, lactic acid concentration and utilised sugar, as shown in Table 4.

The ANOVA table provides convincing evidence that the data exhibited the best fit with a quadratic polynomial model. Out of the four regression models examined, only the quadratic polynomial model demonstrated statistical significance (*p* < 0.05), whereas the other suggested regression models did not exhibit statistical significance (*p* > 0.05). Furthermore, the quadratic model demonstrated strong predictive capabilities, as evidenced by its notably high adjusted R^2^ value (0.8714) and high predicted R^2^ value (0.7323), which were not observed in other suggested regression models.

Moreover, the *p*-value obtained from the lack of fit test conducted on the quadratic polynomial model (0.0502) suggests that the lack of fit is not statistically significant (*p* > 0.05). Therefore, the quadratic polynomial model is deemed suitable for explaining and predicting the selected responses of specific extracellular xylanase activity. This is supported by the good agreement between the predicted and experimental extracellular xylanase activities, as shown in Table 3. The presence of aliased effects between variables was not observed in the quadratic polynomial model, as opposed to the cubic polynomial model. As a result, the quadratic model best describes the relationship between the optimised formulated medium and the specific extracellular xylanase activity of *P. pentosaceus* G4. The following quadratic Equation (2) elucidates the effects of glucose (A), almond shell (B), peanut shell (C), urea (D), and magnesium sulphate (E) on the extracellular xylanase activity of *P. pentosaceus* G4 (Y) in coded symbols (A–E):

(2) $$\begin{array}{l} Y=2.34+0.2789A-0.0071B+0.2408C-0.0709D+0.0026E-0.0768\mathrm{AB}-0.0033\mathrm{AC}-\\ 0.0339\mathrm{AD}-0.0575\mathrm{AE}-0.2069\mathrm{BC}+0.0360\mathrm{BD}-0.0702\mathrm{BE}-0.3298\mathrm{CD}-0.1323\mathrm{CE}-\\ 0.0764\mathrm{DE}-0.2786A^{2}-0.2705B^{2}-0.2549C^{2}-0.3168D^{2}+0.0480E^{2} \end{array}$$

The statistical significance of the quadratic polynomial model of optimised formulated medium and extracellular xylanase production of *P. pentosaceus* G4 was determined using the F-test, and the results are presented in Table 5.

The quadratic polynomial model exhibited a low *p*-value (<0.01), indicating a high level of significance. Additionally, the Model F-value (17.60, *p* < 0.01) suggests that the model is statistically significant. There is only a 0.01% chance that an F-value this large could occur due to noise.

Furthermore, the quadratic polynomial model demonstrated significant predictive capability, effectively accounting for 92% of the variability in the response variable, as evidenced by its high R^2^ value of 0.9239. Likewise, the predicted R^2^ value of 0.7323 and the adjusted R^2^ value of 0.8714 demonstrated an acceptable degree of agreement (difference < 0.2), indicating a robust association between the predicted and experimental values. This suggests that the proposed quadratic polynomial model is statistically significant.

**Figure 2:**
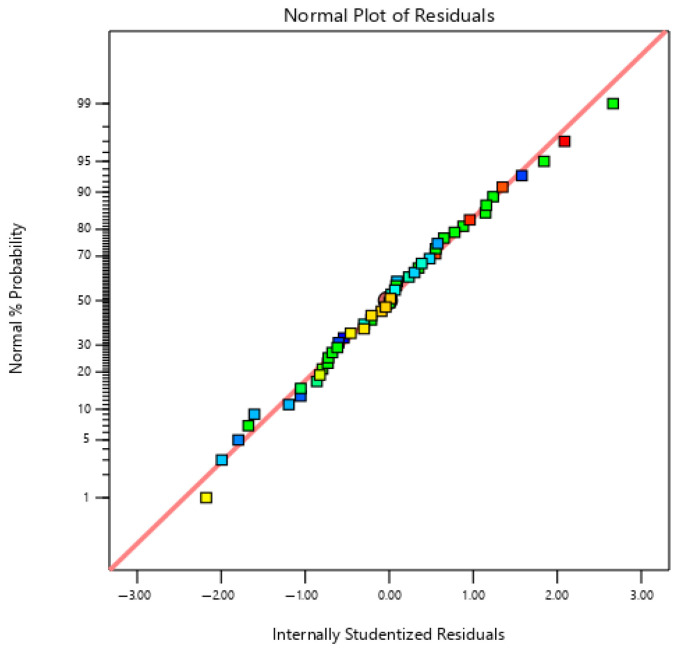
The normality assumption versus internally studentised residuals of the specific extracellular xylanase activity of *P. pentosaceus* G4. The data points are coloured according to specific extracellular xylanase activity, with blue indicating lower specific extracellular xylanase activity and red indicating higher specific extracellular xylanase activity.

The proposed quadratic polynomial model was subsequently subjected to residual analysis, normality testing, and lack-of-fit analysis. The residual analysis was conducted by plotting residuals against predicted values, revealing a random scatter devoid of observable patterns, which confirmed the fulfilment of homoscedasticity and linearity assumptions, as depicted in Figure 2.

**Figure 3:**
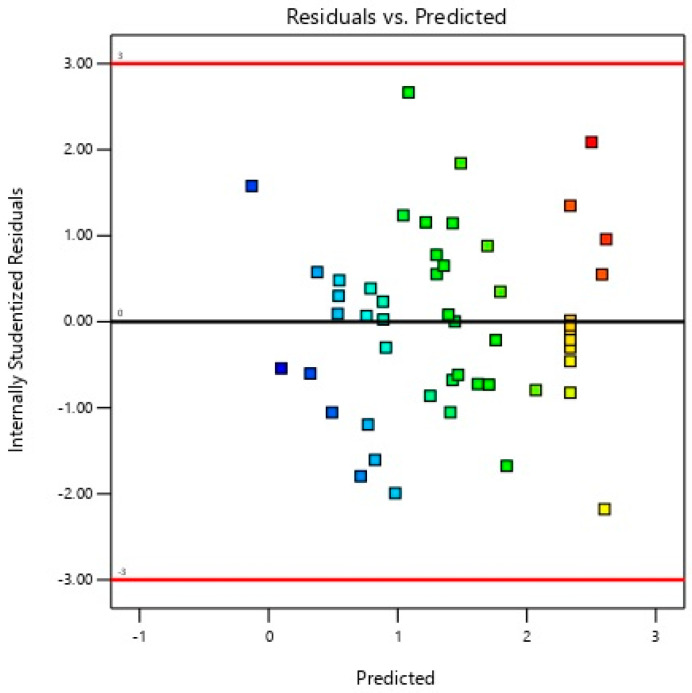
Plot of internally studentised residuals versus predicted values of the specific extracellular xylanase activity of *P. pentosaceus* G4. The data points are coloured according to specific extracellular xylanase activity, with blue indicating lower specific extracellular xylanase activity and red indicating higher specific extracellular xylanase activity.

The adequacy of the normality assumption and the internally studentised residuals were satisfactory, based on the observed straight linear pattern illustrated in Figure 3, hence corroborating the assumption of normal distribution. Furthermore, the lack-of-fit analysis showed no significant lack of fit (*p* > 0.05), indicating that the selected quadratic polynomial models adequately represented the variation in the experimental data. In addition, the quadratic polynomial model demonstrated suitability for navigating the design space due to its sufficient signal-to-noise ratio, as evidenced by the high adequate precision value (15.5135), which far exceeded the fourth threshold value. The validation results collectively demonstrate that the model is statistically stable and suitable for analysing the impact of medium components on extracellular xylanase production of *P. pentosaceus* G4.

Furthermore, the ANOVA results indicate that both the linear and quadratic coefficients of glucose (A) and peanut shell (C) affect extracellular xylanase production significantly (*p* < 0.01). Thus, the interaction coefficients of almond shell and peanut shell (BC), peanut shell and urea (CD), and peanut shell and magnesium sulphate (CE) were subsequently determined for the production of extracellular xylanase by *P. pentosaceus* G4. Surprisingly, there were no significant (*p* > 0.05) interactions between glucose and almond shell (AB), glucose and peanut shell (AC), glucose and urea (AD), glucose and magnesium sulphate (AE), almond shell and urea (BD), almond shell and magnesium sulphate (BE), or urea and magnesium sulphate (DE).

**Figure 4:**
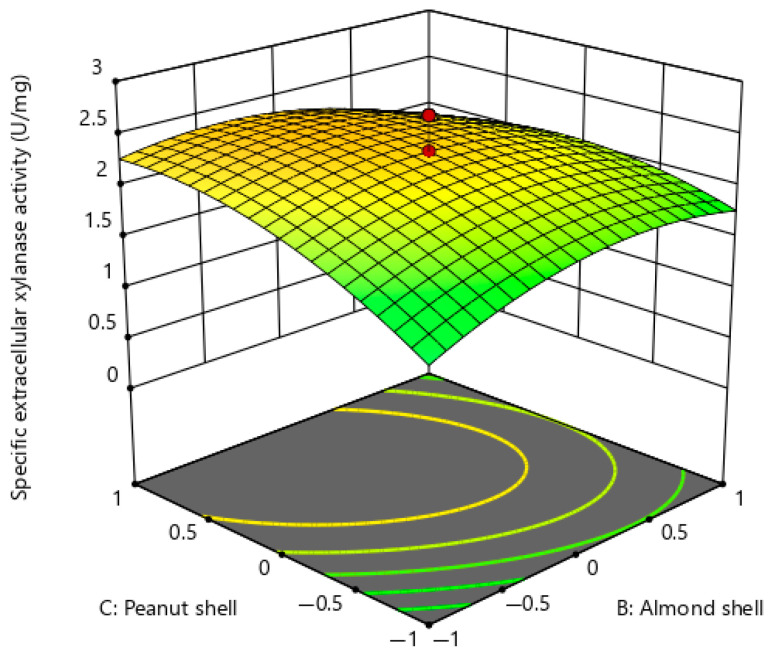
Response surface plot for extracellular xylanase production of *P. pentosaceus* G4 under the interaction of almond shell and peanut shell.

**Figure 5:**
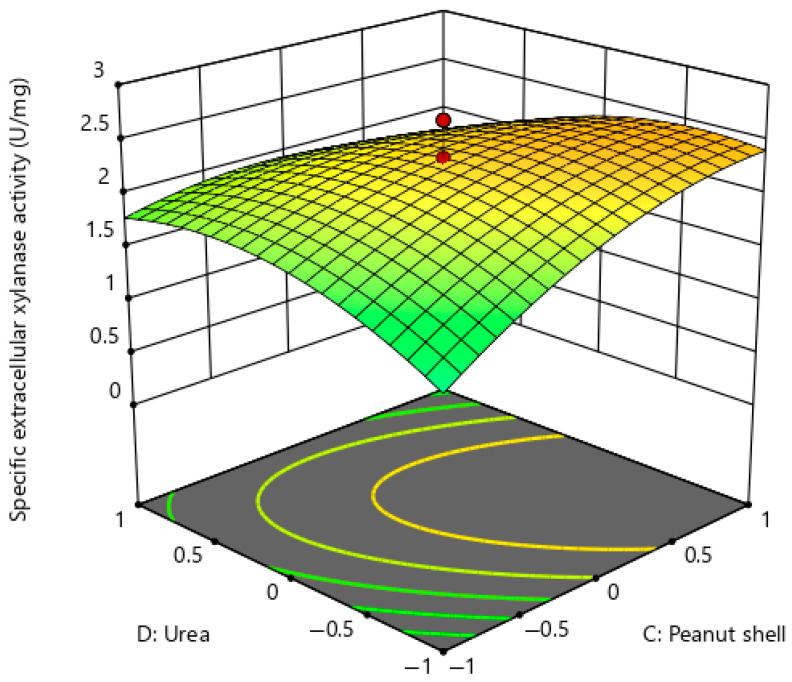
Response surface plot for extracellular xylanase production of *P. pentosaceus* G4 under the interaction of peanut shell and urea.

**Figure 6:**
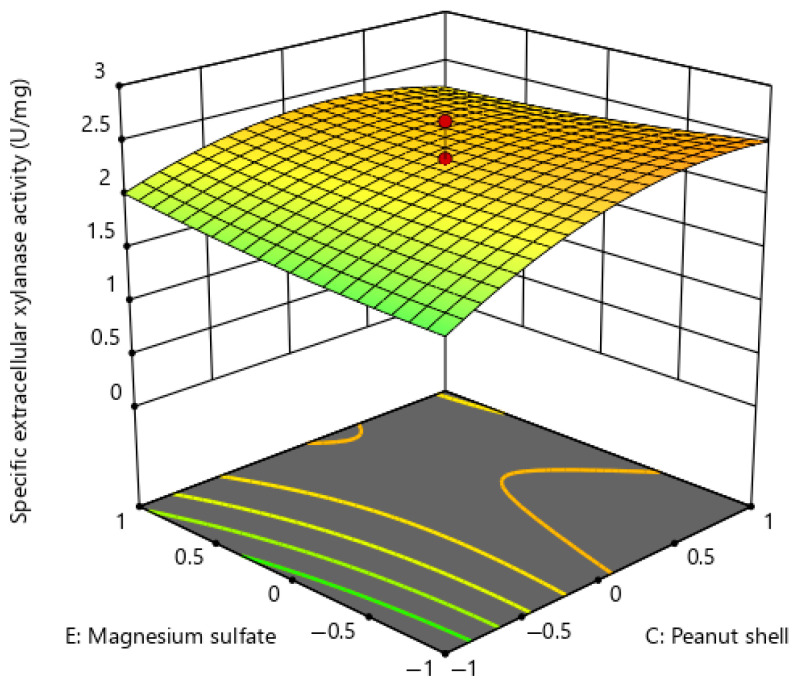
Response surface plot for extracellular xylanase production of *P. pentosaceus* G4 under the interaction of peanut shell and magnesium sulphate.

Response surface plots (Figure 4, Figure 5 and Figure 6) were constructed to confirm the interaction effects of essential medium components on extracellular xylanase production of *P. pentosaceus* G4. Figure 4 illustrates the interaction between two carbon sources, namely almond shell and peanut shell, while maintaining glucose, urea, and magnesium sulphate at a constant concentration of 20 g/L, 4 g/L, and 0.2 g/L, respectively. The response surface plot demonstrates a pronounced upward surface curvature, indicating an improvement in the production of extracellular xylanase when the concentration of almond shell and peanut shell increased concurrently within the examined range, hence implying a synergistic interaction that positively influenced extracellular xylanase production. Furthermore, the maximum extracellular xylanase production was noted when the almond shell concentration was between 10 g/L and 20 g/L, while the peanut shell concentration was between 20 g/L and 30 g/L. The interaction model of almond shell and peanut shell showed a statistically significant effect (BC), yielding a *p*-value < 0.01 (Table 5).

Figure 5 illustrates the reaction between peanut shell (a carbon source) and urea (a nitrogen source), with glucose (20 g/L), almond shell (20 g/L), and magnesium sulphate (0.2 g/L) maintained at constant levels. The response surface plot displays a convex configuration, with the peak response identified at elevated levels of both peanut shell and urea. The curvature and gradient suggest a synergistic effect of peanut shell and urea. The production of extracellular xylanase was optimised when the concentration of peanut shells was between 20 g/L and 30 g/L, and the range of urea substrate was from 2 g/L to 4 g/L. Table 5 shows that the interaction model of peanut shell and urea has a statistically significant effect, since the interaction of peanut shell and urea (CD) produces a *p*-value of less than 0.01.

The response surface plot of Figure 6 depicts the interaction between peanut shell and magnesium sulphate (CE) on extracellular xylanase production. The concentrations of glucose, almond shell, and urea were maintained at their centre values of 20 g/L, 20 g/L, and 4 g/L, respectively. The response surface plot displays a slightly curved plane, signifying a non-linear correlation between the two medium factors. An elevation in the concentrations of both peanut shell and magnesium sulphate resulted in improved extracellular xylanase synthesis. An optimal output was attained when the peanut shell concentration ranged from 20 g/L to 30 g/L, and the magnesium sulphate concentration remained at a comparatively low level of 0.1 g/L. This indicates a threshold effect, where additional magnesium sulphate may not enhance production and could potentially inhibit the production of extracellular xylanase. The ANOVA results (Table 5) indicate a statistically significant interaction between peanut shell and magnesium sulphate (CE) on extracellular xylanase production (*p* < 0.05).

CCD of RSM predicted extracellular xylanase production of 2.737 U/mg with the optimised growth medium by *P. pentosaceus* G4, which is similar to the experimental specific extracellular xylanase activity of 2.7646 U/mg. In comparison, the MRS commercial medium yielded a specific extracellular xylanase activity of 0.8809 U/mg, with a medium cost of 11.11 USD/L. Following medium optimisation, the specific extracellular xylanase activity increased 3.13-fold (2.7646 U/mg), with the price of the optimised medium concurrently decreasing to 1.391 USD/L, reflecting a 7.99-fold reduction in medium expenses, as shown in Table 6. The results suggest that the optimised formulated growth medium is a cost-effective alternative to the MRS commercial medium for extracellular xylanase production of *P. pentosaceus* G4, thereby enhancing the process’s viability for industrial applications.

Statistical optimisation is a highly effective and versatile approach for determining the optimal values for process parameters to maximise the intended product yield [25]. The findings of this study align well with those of other CCD studies on xylanase production reported for various bacteria. Coman and Bahrim [24] reported that the CCD approach increased the xylanase production of *Streptomyces* sp. P12-137 by 3-fold with wheat bran as the substrate, whereas Bibra et al. [25] demonstrated that the CCD increased the production of xylanase of *Geobacillus* sp. strain DUSELR13 from 6 U/mL to 31 U/mL, using the lignocellulosic biomass of prairie cordgrass and maize stover as the substrate. Furthermore, Kaushal, Sharma, and Dogra [92] employed the CCD approach to increase cellulase-free endo-β-1,4-xylanase production of *Bacillus subtilis* SD8 to 8.18 IU/mL. In comparison, Patel and Dudhagara [93] observed a twofold enhancement (19.46 U/mL) in the production of xylanase of *Bacillus tequilensis* strain UD-3 after optimisation with CCD, whereas Sharma, Sharma, and Mahajan [94] conducted a study on *Bacillus subtilis* SD8 that exhibited optimised xylanase activity of 8.18 IU/mL.

The results of this study highlight the effectiveness of PBD and CCD statistical methods in assessing and optimising the medium composition that affects extracellular xylanase production of *P. pentosaceus* G4. Notably, the optimised formulated medium showed a significant increase in extracellular xylanase levels compared to the non-optimised medium. This indicates the potential practicality of utilising *P. pentosaceus* G4 as a viable and influential producer of extracellular xylanase using renewable agro-biomass polymers, such as nutshell. Additionally, the findings of this study demonstrated the potential of utilising extracellular xylanase-producing LAB as a safe alternative for industrial xylanase production. Despite this finding making a valuable contribution to the field of industrial enzyme manufacturing, it also introduces the vast potential of LAB in producing extracellular hemicellulolytic enzymes.

#### 2.2.2. Correlation of Other Factors Associated with Extracellular Xylanase Production of *P. pentosaceus* G4

In the current study, the correlation between extracellular xylanase production, cell population, lactic acid production, sugar utilisation, and pH reduction in the growth medium of *P. pentosaceus* G4 strains has been investigated. Addressing the intricate relationship among these variables is crucial for maximising the yield of extracellular xylanase of the producer strain.

##### Cell Viability of *P. pentosaceus* G4

During the investigation of the impact of medium components on the growth of *P. pentosaceus* G4 via CCD experimental runs, it was noted that the maximum cell population of *P. pentosaceus* G4 was 8.3531 Log CFU/mL, which was obtained in experimental run 22. The observed growth demonstrated statistical significance (*p* < 0.05) in comparison to other experimental runs. The control MRS medium only yielded a cell population of 7.8273 Log CFU/mL. The experimental run 22 also demonstrated the highest specific extracellular xylanase activity, implying a distinct correlation between the highest growth of *P. pentosaceus* G4 and the highest production of the extracellular xylanase enzyme. Table 7 describes the potential regression models for defining the relationship between the medium components and the cell population of *P. pentosaceus* G4.

Based on the results of the ANOVA, it can be concluded that the quadratic polynomial model is the best option, as it exhibits statistical significance (*p* < 0.05), an adjusted R^2^ value of 0.7790, and a predicted R^2^ value of 0.5415, among the four regression models. Furthermore, it lacks any form of aliasing effects among variables. The quadratic polynomial model demonstrates a strong fit with the response variable. The lack of fit test gave a non-significant *p*-value of 0.2045, indicating that there is no significant lack of fit in the quadratic polynomial model. The quadratic Equation (3) describes the impact of glucose (A), almond shell (B), peanut shell (C), urea (D), and magnesium sulphate (E) on the cell population of *P. pentosaceus* G4 (Z) using coded symbols (A–E):

(3) $$\begin{array}{l} Z=8.15+0.0468A+0.0009B+0.0459C-0.0071D+0.0109E-0.0091\mathrm{AB}-0.0011\mathrm{AC}-\\ 0.0110\mathrm{AD}-0.0129\mathrm{AE}-0.0357\mathrm{BC}+0.0091\mathrm{BD}-0.0157\mathrm{BE}-0.0565\mathrm{CD}-0.0181\mathrm{CE}-\\ 0.0175\mathrm{DE}-0.0398A^{2}-0.0342B^{2}-0.0364C^{2}-0.0493D^{2}+0.0125E^{2} \end{array}$$

The statistical significance of the quadratic polynomial model for the optimised formulated medium and cell population of *P. pentosaceus* G4 was determined using the F-test, and the results are presented in Table 8.

The model demonstrated a statistically significant result with a *p*-value of less than 0.0001, suggesting a high level of significance at a threshold of *p* < 0.01. Moreover, based on the Model F-value (*p* < 0.01), it can be inferred that the model has statistical significance. There is only a 0.01% chance that an F-value this large could occur due to noise. In addition, it is worth noting that the model exhibited a significant level of predictive capacity, successfully explaining 86% of the variation in the dependent variable, as indicated by its notable R^2^ value of 0.8692, predicted R^2^ of 0.5415, and adjusted R^2^ of 0.7790. The F-value of 1.85 for the Lack of Fit indicates that when evaluated against the pure error, the Lack of Fit does not reach statistical significance.

Moreover, the quadratic polynomial model demonstrated its suitability for navigating the design space due to its adequate signal-to-noise ratio. This is supported by the high adequate precision value (13.1801). However, the ANOVA results indicate that the linear and quadratic coefficients of glucose (A) and peanut shell (C), as well as the interaction between the almond shell and peanut shell (BC) and peanut shell and urea (CD), were observed to have a significant contribution (*p* < 0.05) to the cell population of *P. pentosaceus* G4. In contrast, the remaining interactions, namely glucose and almond shell (AB), glucose and peanut shell (AC), glucose and urea (AD), glucose and magnesium sulphate (AE), almond shell and urea (BD), almond shell and magnesium sulphate (BE), peanut shell and magnesium sulphate (CE), and urea and magnesium sulphate (DE) did not exhibit any statistically significant differences (*p* > 0.05).

**Figure 7:**
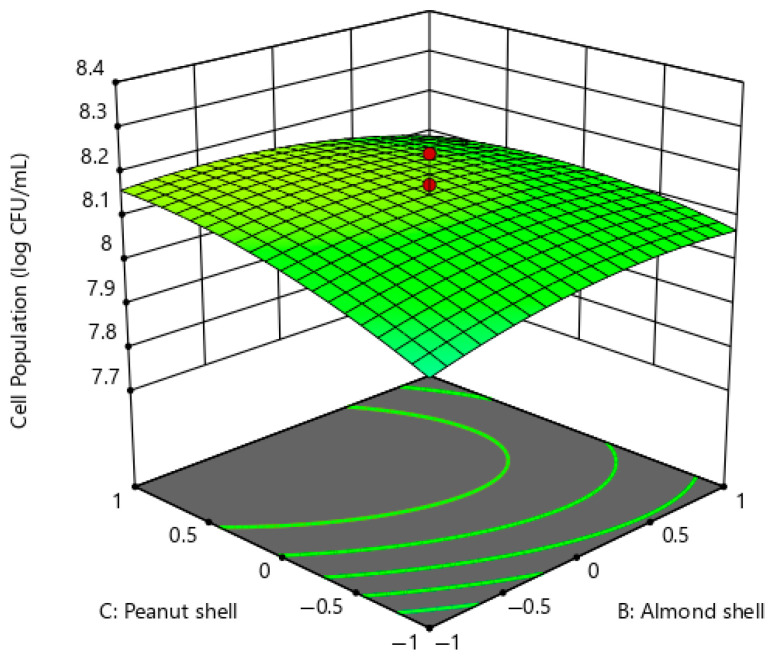
Response surface plot of *P. pentosaceus* G4 cell population under the interactions of almond shell and peanut shell.

Figure 7 illustrates the interaction between the almond shell and the peanut shell on the cell population of *P. pentosaceus* G4. The concentrations of glucose, urea, and magnesium sulphate were fixed at the central point of 20 g/L, 4 g/L, and 0.2 g/L, respectively. The response surface plot displays a positive gradient along both axes, indicating a synergistic interaction between the two carbon sources of the medium components. This showed that *P. pentosaceus* G4 could mobilise the hemicellulose-rich nutrient content of almond and peanut shells via metabolic activities for its growth and other physiological functions. The maximum cell population of *P. pentosaceus* G4 was achieved with almond shell concentrations from 10 g/L to 20 g/L, while peanut shell concentrations ranged from 20 g/L to 30 g/L. The growth of *P. pentosaceus* G4 was inhibited when the concentration of either the almond shell or the peanut shell was beyond or under the stated concentrations.

**Figure 8:**
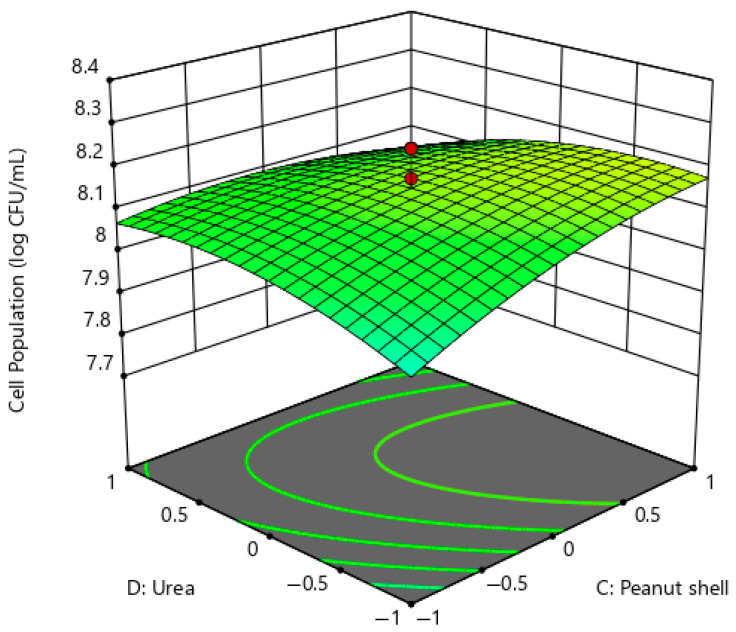
Response surface plot of *P. pentosaceus* G4 cell population under the interactions of peanut shell and urea.

Figure 8 illustrates the three-dimensional surface plot of the *P. pentosaceus* G4 cell population as a function of peanut shell and urea while maintaining glucose, almond shell, and magnesium sulphate concentrations at the central values of CCD of 20 g/L, 20 g/L, and 0.2 g/L, respectively. The surface plot illustrates a gradually ascending surface profile, suggesting that the directed response endorses a cooperative effect. The augmentation of the peanut shell and urea concentration enhanced the growth of *P. pentosaceus* G4. The maximum cell population was observed when the peanut shell concentration was 30 g/L, while the urea concentration varied between 0 g/L and 2 g/L. The optimal concentrations of glucose, almond shell, peanut shell, urea, and magnesium sulphate were suggested to be 26.537 g/L, 10.406 g/L, 30 g/L, 2.015 g/L, and 0.300 g/L, respectively, with a predicted cell population of 8.241 Log CFU/mL. The statistical model was validated by culturing *P. pentosaceus* G4 in the recommended optimal medium, resulting in a cell population of 8.2495 Log CFU/mL, which was similar to the predicted cell population of 8.241 Log CFU/mL. The cell population of *P. pentosaceus* G4 in the optimised formulated medium increased by approximately 1.05-fold compared to the MRS commercial medium (7.8273 Log CFU/mL).

The current study reveals the significant influence of glucose on the growth of *P. pentosaceus* G4, with the cell population exceeding 8.2 log CFU/mL. Glucose, a primary carbon substrate for LAB catabolic activities, initiates the glycolysis metabolic pathway, generating metabolic byproducts that contribute to the rapid growth of the microbial cell [54]. This is in agreement with Lim et al. [17] on the growth of *P. acidilactici* TP-6, highlighting the crucial role of the carbon source in supporting the survival and growth of producer strains. Lim et al. [91] and Zabidi et al. [30] subsequently observed that media containing glucose would enhance the growth of *Pediococcus pentosaceus* TL-3 and *Lactobacillus plantarum* RI 11, respectively. However, it is noteworthy that higher glucose concentrations could inhibit LAB growth [95].

The depletion of glucose and subsequent fall in pH would decrease the number of glycolytic intermediates, hence compelling LAB to utilise other carbon sources through heterofermentation [95]. According to Jiang et al. [96], multiple strains of *P. pentosaceus* can utilise a wide range of carbon sources effectively. Nevertheless, limited information is available on the growth of *P. pentosaceus* on agro-waste that consists of a diverse array of sugars, lignin, hemicelluloses and cellulose [9]. Renewable agro-waste biomass has promising potential as an inexpensive and sustainable source of carbon and nutrient resources for the growth of various microorganisms, facilitating the production of metabolites. The present study demonstrated the notable and significant impact of peanut shells on the growth of *P. pentosaceus* G4, which aligned with the findings of Lim et al. [17] on the stimulated growth of *P. acidilactici* TP-6 with molasses agro-waste. Zabidi et al. [30] reported that *Lactiplantibacillus plantarum* (formerly known as *Lactobacillus plantarum)* RI 11 exhibited the maximum cell biomass production (log 10.51 CFU/mL) when the growth medium was supplemented with molasses.

LAB are characterised as fastidious microorganisms that are incapable of growing on a mineral substrate supplied with only a carbon source. According to Raman et al. [97], acidogenic microorganisms such as LAB require a complete medium containing nitrogen sources attributed to their restricted amino acid anabolic metabolic pathways. Therefore, amino acids, peptides, vitamins, and minerals are essential for the growth of LAB [98]. Interestingly, in the present study, urea did not significantly impact the cell population of *P. pentosaceus*, which aligns with the results of Lim et al. [91] reported on the effect of urea on the cell population of *P. pentosaceus* TL-3. According to Carvalho et al. [99], the addition of urea resulted in an elevation in pH without exhibiting any stimulating effects on the growth of LAB, which was in contrast to the significant interaction between urea and peanut shells obtained in this study.

As for the effect of minerals, the present study did not observe a statistically significant impact of MgSO_4_ on the cell population of *P. pentosaceus* G4. This finding contradicts the results reported by Lim et al. [91], who found that MgSO_4_ significantly influenced the cell growth of *P. pentosaceus* TL-3. However, our findings on the effects of MgSO_4_ are consistent with the research conducted by Giridhar and Chandra [45], who also reported that MgSO_4_ exhibited a minor inhibitory effect on *Gracilibacillus* sp. TSCPVG. It is worth mentioning that the influence of MgSO_4_ on the growth of *Pediococcus* sp. depends on the microorganism’s metabolic traits and tolerance, as indicated by the limited growth enhancement reported in *Pediococcus damnosus* [100]. This discrepancy in results highlights the variation in the requirement of MgSO_4_ by different bacterial cells, emphasising the need for a nuanced understanding of strain-specific responses to MgSO_4_ supplementation.

The present study reveals that the production of extracellular xylanase enzymes corresponded to the growth of *P. pentosaceus* G4. Understanding the correlation between bacterial growth and the production of extracellular xylanase could contribute to strategies aimed at improving microbial growth and enhancing enzyme production for various biotechnological purposes.

##### Lactic Acid Production and pH

The experimental run that exhibited the highest lactic acid production was experimental run 22, with approximately 17.3171 g/mL of lactic acid. However, the lactic acid concentration of the control MRS medium was 18.5889 g/mL, which was significantly higher (*p* < 0.05) than the amount of lactic acid produced in experimental run 22. Although *P. pentosaceus* G4 produced slightly higher (1.2718 g/mL) lactic acid in MRS commercial medium, a comparable amount of lactic acid concentration of 17.3171 g/mL was produced in optimised formulated medium, implying that *P. pentosaceus* G4 could adaptively catabolise almond and peanut shells containing xylan to lactic acid via heterofermentative pathways [95].

The results of the current study align with previous research. Karne and Moharir [101] reported a maximum production of 0.80 g lactic acid/g using *Rhizopus oryzae*. Likewise, Brown, Grunden, and Pawlak [102] employed CCD to enhance the lactic acid production of *Paenibacillus glucanolyticus* SLM1 to 0.26 g/L with lignocellulosic substrate. In a parallel context, Altaf, Naveena, and Reddy [103] demonstrated lactic acid production of *Lactobacillus amylophilus* GV6 with red lentil and Baker’s yeast cells, alongside starch, which resulted in a maximum lactic acid production of 13.5 g. Interestingly, Katepogu et al. [104] reported a lactic acid yield of 56.5 g/L when banana crop residue was used to grow *Pediococcus pentosaceus* HLV1, whereas Coelho et al. [105] attained a maximal lactic acid production of 94.8 g/L from molasses by *Lactobacillus plantarum* LMISM6. Substantially higher lactic acid production of 121 g/L was shown by Wang et al. [106] via a mixed fermentation approach with *Lactobacillus rhamnosus* and *Bacillus coagulans* using sweet sorghum juice. In comparison, the present study achieved a moderate quantity of lactic acid production by *P. pentosaceus* G4, which adaptively catabolises almond and peanut shells containing predominantly hemicellulose xylan, suggesting that *P. pentosaceus* G4 can transform eco-friendly, renewable agro-waste biomass polymers of almond and peanut shells into lactic acid. This biotransformation process correlates positively with extracellular xylanase production of *P. pentosaceus* G4, contributing to the reduction of agro-waste and pollution prevention through valorisation, while simultaneously creating wealth by transforming it into value-added products, such as lactic acid.

The accumulation of lactic acid reduces the pH of the cultivation medium, as demonstrated in this study, where the pH decreased from 6.23 to 4.21, creating an acidic environment, a signature characteristic of LAB. The reduction in pH acts as a stimulus for the activation of genes involved in the synthesis of extracellular xylanase, which plays a vital role in regulating the production of extracellular xylanase of *P. pentosaceus* G4.

##### Sugar Utilisation

Despite experimental run 22 exhibiting the most significant utilisation of sugar (13.1090 g/L) among the other experimental runs, the utilised sugar in this run was lower than that observed with the control MRS medium (14.0538 g/L), suggesting that the different metabolic pathways of *P. pentosaceus* G4 associated with sugar consumption could be activated under different medium compositions, since there was not a significant increase in sugar utilisation under the optimal formulated medium condition. It could be attributed to the adaptive shift to xylan-containing renewable biopolymers, accompanying by the production of extracellular xylanase enzymes, which signifies a change in adaptive catabolic activity to utilise xylan as an alternative carbon source instead of glucose.

Glucose has a simple structure that can be catabolised simply via fermentation, to support microbial growth [107,108]. Sugar utilisation of *P. pentosaceus* G4 was likely to be influenced by the availability of glucose, which is a preferred substrate. The process of glucose absorption and conversion into glucose-6-phosphate is a crucial step in the glycolysis pathway. This metabolic pathway enables the generation of ATP and NADH, which play a vital role in supporting microbial growth and facilitating the synthesis of essential metabolites [109,110]. Nonetheless, the inclusion of glucose in the medium could induce carbon catabolite repression, thereby suppressing enzyme excretion through the inhibition of gene expression related to the catabolism of the alternate carbon source [111]. The quantity of sugar used for xylanase production can vary, depending on several factors, including the bacterial strain, substrate composition, substrate concentration, and enzyme efficacy. A study carried out by Farliahati et al. [112] found that *Escherichia coli* DH5α exhibited a xylanase production of 2649 U/mL when cultivated with a glucose concentration of 15 g/L as the substrate. A separate investigation was conducted by Alokika and Singh [113], which specifically examined the xylanases of *Bacillus substilis* subsp. subtilis JJBS250 and *Myceliophthora thermophila* BJTLRMDU3 using untreated sugarcane bagasse that contained reducing sugars of 124.24 mg/g as substrate.

Glucose has different effects on sugar usage and extracellular xylanase synthesis of *P. pentosaceus* G4. The positive interaction between the consumption of sugar, the growth of *P. pentosaceus* G4, and the subsequent production of extracellular xylanase demonstrated that *P. pentosaceus* G4 could efficiently produce extracellular xylanase to catabolise the renewable biomass polymers of almond and peanut shells in the optimised cost-effective formulated medium (Table 6). Overall, the production of extracellular xylanase of *P. pentosaceus* G4 is positively correlated with cell population, lactic acid concentration, the amount of sugar utilised and pH, indicating multifaceted interactions among physiological variables that influence extracellular xylanase production.

## 3. Materials and Methods

### 3.1. Inoculum Maintenance and Preparation

*P. pentosaceus* G4 was previously isolated from the gundelia (*Gundelia tournefortii*) plant. It was employed as the bacterial cells for extracellular xylanase production in this study. The pure stock culture was preserved in MRS broth (Neogen Co., Lansing, MI, USA) containing 20% (*v*/*v*) glycerol (Merck, Darmstadt, Germany) and kept at −30 °C. The active *P. pentosaceus* G4 was prepared by reviving the stock culture with MRS broth [114], rinsing it once with a sterile solution of 0.85% (*w*/*v*) NaCl (Pharmacia, Uppsala, Sweden), followed by adjusting the optical density at 600 nm to 1, as described by Lee et al. [29], to prepare a log 9 CFU/mL cell population of *P. pentosaceus* G4 to be used as the inoculum for subsequent experiments.

### 3.2. Experimental Design of Optimisation of Extracellular Xylanase Production

The optimisation of extracellular xylanase production of *P. pentosaceus* G4 was mediated by the PBD and CCD of the RSM statistical approach. The PBD was initially employed to identify the effect of each medium component on the production of extracellular xylanase of *P. pentosaceus* G4. Subsequently, the concentrations of the positive medium components, as suggested by PBD analyses, were optimised using the CCD. The experimental design and statistical data analysis of both PBD and CCD were conducted using Design-Expert statistical software version 13 (State-Ease Inc., Minneapolis, MN, USA).

#### 3.2.1. Plackett–Burman Design

In the present study, 19 medium component variables were selected based on the medium composition of the commercial MRS medium, which served as the control medium. Additionally, a few nutshells were used together with the other components of the MRS medium. Each medium component was set with a coded value of a low concentration level (−1) and a high concentration level (+1) to assess the effect of the medium component on extracellular xylanase production of *P. pentosaceus* G4, as shown in Table 9. The PBD has suggested 20 experimental runs with various combinations and concentrations of the 19 medium components, as shown in Table 10.

PBD suggested the following equation to evaluate the 19 medium component variables in relation to the specific extracellular xylanase activity responses, thereby reflecting the extracellular xylanase production of *P. pentosaceus* G4. Equation (4) illustrates the first-order model equation.

(4) $$\Upsilon =\beta_{0}+\sum_{n=1}^{20}\;\beta_{i\;}+X_{i,}$$

where Υ represents the specific extracellular xylanase activity responses (variables), *β*_0_ is the intercept coefficient, and *β_i_* is the coefficient for the linear effects of the independent variables (X_1_ − X_20_).

#### 3.2.2. Central Composite Design

The CCD was employed to determine the optimal concentrations of five positive medium components (verified via PBD, Section 3.3) of glucose, almond shell, peanut shell, urea, and magnesium sulphate that significantly affected the extracellular xylanase production of *P. pentosaceus* G4. Each medium component variable was assigned to five concentration levels, represented by the symbols +α, +1, 0, −1 and -α, as shown in Table 11.

The CCD proposed 50 experimental runs, as shown in Table 12, comprising 32 factorial points, 10 axial points, and 8 central points. The experiments were carried out simultaneously, encompassing the entire spectrum of possible combinations of various concentrations of each positive medium component for extracellular xylanase production of *P. pentosaceus* G4.

CCD suggested the following equation to evaluate the correlations between the 5 positive medium component variables and the specific extracellular xylanase activity responses, reflecting extracellular xylanase production of *P. pentosaceus* G4. Equation (5) illustrates the second-order model equation.

(5) $$\Upsilon =\beta_{0}+\sum \;\beta_{i\;}X_{i,}+\sum \;\beta_{j^{2}}\;X_{j^{2}}+\sum \;\beta_{jk\;}X_{j}\;X_{k}$$

where Υ represents the response variable, the symbol *β*_0_ represents the coefficient of interception, *β_i_* represents the linear coefficients, *β_j_*^2^ represents the quadratic coefficients, and *β_jk_* represents the interactive coefficients.

### 3.3. Extracellular Xylanase Production of P. pentosaceus G4

The almond shell, peanut shell, hazelnut shell, pistachio shell, and walnut shell were the renewable agro biopolymers that were dried and ground into a fine powder using an electric grinder [3] as the medium components (Table 10 and Table 12) for the extracellular xylanase production by *P. pentosaceus* G4. A 10% (*v*/*v*) log 9 CFU/mL active inoculum (Section 3.1) of *P. pentosaceus* G4 was added to 100 mL of the respective medium combination (Table 10 and Table 12) in a 250 mL Erlenmeyer flask and incubated at 30 °C for 24 h without agitation [30]. Centrifugation was performed at 10,000× *g*, for 15 min at 4 °C to collect the cell-free supernatant (CFS), followed by filtration using a cellulose acetate membrane [29,115]. The CFS was used to determine the specific extracellular xylanase enzyme activity, lactic acid concentration, final pH, and utilised sugar concentration.

### 3.4. Determination of Cell Viability

The cell viability of *P. pentosaceus* G4 was quantified using the Standard Plate Count method. A volume of 1 mL of bacterial culture was collected from each growth medium, as suggested by CCD (Table 12) for determining cell viability. The cell pellet was collected by centrifugation at 10,000 × *g* for 15 min at 4 °C. The cell pellet was washed with 1 mL of 0.85% (*w/v*) NaCl. Subsequently, the washed cell pellet was resuspended by vortexing (Stuart Scientific, Stafford, UK) for 3 min in 1 mL of 0.85% (*w*/*v*) NaCl solution. A 10-fold serial dilution was performed using a 0.85% (*w*/*v*) NaCl solution for the bacterial cell suspension. A volume of 100 μL from the appropriately diluted bacterial cell suspensions was evenly spread onto MRS agar plates (Neogen Co., Lansing, MI, USA) and incubated in an anaerobic incubator at 30 °C for 48 h [116].

### 3.5. Determination of Lactic Acid Concentration

The lactic acid concentration of the collected CFS was determined using the methodology outlined by Borshchevskaya et al. [117]. In brief, 1 mL of 0.2% FeCl_3_ was mixed well with 25 μL of the collected CFS. The measurement of absorbance at 360 nm was performed within 15 min after mixing the reagent. A reference of lactic acid was constructed for the determination of the lactic acid concentration of CFS.

### 3.6. Determination of Sugar Utilisation

Sugar utilisation was determined by measuring the reducing sugar concentration in the growth medium before and after fermentation using the dinitrosalicylic acid (DNS) method, as described by Miller [118]. The difference between the reducing sugar concentration of the growth medium before and after fermentation indicates the amount of sugar utilised. A glucose solution was used to construct the reducing sugar reference for determining sugar utilisation.

### 3.7. Initial and Final pH Determination

The pH was monitored using a pH metre (Jenway, Stone, UK) at the beginning and end of the fermentation process.

### 3.8. Statistical Analysis

All experiments were performed in triplicate. The results were presented as a mean ± the standard error of the mean (SEM). The statistical analysis was performed using R Studio software (version 2023.09.1 + 494).

## 4. Conclusions

In conclusion, an extensive investigation has revealed that *P. pentosaceus* G4 exhibits a high capacity for producing extracellular xylanase enzymes when cultivated in an optimised formulated medium comprising cost-effective and environmentally friendly renewable biopolymers. The PBD statistical approach has revealed that 8 out of the 19 tested medium components, including glucose, almond shell, peanut shell, walnut shell, malt extract, xylan, urea and magnesium sulphate, had a positive and significant impact (*p* < 0.05) on the production of extracellular xylanase of *P. pentosaceus* G4. The extracellular xylanase production was significantly negatively affected (*p* < 0.05) by the other medium components, including hazelnut shell, pistachio shell, peptone, yeast extract, meat extract, sodium acetate and dipotassium hydrogen phosphate. However, only glucose, peanut shell, urea, magnesium sulphate and almond shell were selected for the subsequent concentration optimisation via the CCD, attributed to their highly significant effect on extracellular xylanase production of *P. pentosaceus* G4. The CCD has suggested an optimised formulated medium comprising glucose (26.87 g/L), almond shell (16 g/L), peanut shell (30 g/L), urea (2.85 g/L) and magnesium sulphate (0.10 g/L) to have a predicted specific extracellular xylanase activity of 2.737 U/mg, which was similar to the experimental specific extracellular xylanase activity of 2.7646 U/mg. The optimised formulated medium has resulted in a significant 3.13-fold enhancement in the production of extracellular xylanase, while decreasing the cost of the fermentation medium substantially to 7.99-fold compared to the commercial MRS medium. The highest extracellular xylanase activity was observed at 2.9243 U/mg in the experimental run of 22, which coincided with the highest cell population of 8.3531 log CFU/mL, the highest concentration of lactic acid production of 17.3171 g/mL and the highest amount of sugar utilisation of 13.1090 g/L. Hence, the final pH was recorded at 4.21. The results obtained in this study have revealed the vast potential of *P. pentosaceus* G4 as an extracellular xylanase producer using the optimised formulated medium comprising renewable agro biomass.

## Figures and Tables

**Table 1:** Specific extracellular xylanase activity of *P. pentosaceus* G4 corresponding to the experimental run of Plackett–Burman Design.

Experimental Run	A	B	C	D	E	F	G	H	J	K	L	M	N	O	P	Q	R	S	T	Specific Extracellular Xylanase Activity (U/mg)
1	1	1	−1	−1	1	1	1	1	−1	1	−1	1	−1	−1	−1	−1	1	1	−1	0.2913 ^cd^ ± 0.0295
2	−1	1	1	−1	−1	1	1	1	1	−1	1	−1	1	−1	−1	−1	−1	1	1	0.6062 ^b^ ± 0.0497
3	1	−1	1	1	−1	−1	1	1	1	1	−1	1	−1	1	−1	−1	−1	−1	1	0.2570 ^d^ ± 0.0442
4	1	1	−1	1	1	−1	−1	1	1	1	1	−1	1	−1	1	−1	−1	−1	−1	0.1289 ^efg^ ± 0.0381
5	−1	1	1	−1	1	1	−1	−1	1	1	1	1	−1	1	−1	1	−1	−1	−1	0.0135 ^i^ ± 0.0024
6	−1	−1	1	1	−1	1	1	−1	−1	1	1	1	1	−1	1	−1	1	−1	−1	0.0121 ^i^ ± 0.0037
7	−1	−1	−1	1	1	−1	1	1	−1	−1	1	1	1	1	−1	1	−1	1	−1	0.1552 ^ef^ ± 0.0133
8	−1	−1	−1	−1	1	1	−1	1	1	−1	−1	1	1	1	1	−1	1	−1	1	0.1071 ^efg^ ± 0.0086
9	1	−1	−1	−1	−1	1	1	−1	1	1	−1	−1	1	1	1	1	−1	1	−1	0.1207 ^efg^ ± 0.0050
10	−1	1	−1	−1	−1	−1	1	1	−1	1	1	−1	−1	1	1	1	1	−1	1	0.0989 ^fgh^ ± 0.0620
11	1	−1	1	−1	−1	−1	−1	1	1	−1	1	1	−1	−1	1	1	1	1	−1	0.3486 ^c^ ± 0.0098
12	−1	1	−1	1	−1	−1	−1	−1	1	1	−1	1	1	−1	−1	1	1	1	1	0.0000 ^i^ ± 0.0000
13	1	−1	1	−1	1	−1	−1	−1	−1	1	1	−1	1	1	−1	−1	1	1	1	0.0741 ^ghi^ ± 0.0058
14	1	1	−1	1	−1	1	−1	−1	−1	−1	1	1	−1	1	1	−1	−1	1	1	0.1817 ^e^ ± 0.0046
15	1	1	1	−1	1	−1	1	−1	−1	−1	−1	1	1	−1	1	1	−1	−1	1	0.6453 ^b^ ± 0.0382
16	1	1	1	1	−1	1	−1	1	−1	−1	−1	−1	1	1	−1	1	1	−1	−1	1.0329 ^a^ ± 0.0307
17	−1	1	1	1	1	−1	1	−1	1	−1	−1	−1	−1	1	1	−1	1	1	−1	0.0278 ^hi^ ± 0.0039
18	−1	−1	1	1	1	1	−1	1	−1	1	−1	−1	−1	−1	1	1	−1	1	1	0.0000 ^i^ ± 0.0000
19	1	−1	−1	1	1	1	1	−1	1	−1	1	−1	−1	−1	−1	1	1	−1	1	0.0000 ^i^ ± 0.0000
20	−1	−1	−1	−1	−1	−1	−1	−1	−1	−1	−1	−1	−1	−1	−1	−1	−1	−1	−1	0.0000 ^i^ ± 0.0000

Notes: +1 and −1 indicate the lowest and highest values of each nutrient variable. A: Glucose (0 g/L–20 g/L); B: Almond shell (0 g/L–20 g/L); C: Peanut shell (0 g/L–20 g/L); D: Hazelnut shell (0 g/L–20 g/L); E: Pistachio shell (0 g/L–20 g/L); F: Walnut shell (0 g/L–20 g/L); G: Malt extract (0 g/L–5 g/L); H: Xylan (0 g/L–20 g/L); J: Peptone (0 g/L–10 g/L); K: Yeast extract (0 g/L–4 g/L); L: Meat extract (0 g/L–8 g/L); M: Ammonium citrate (0 g/L–2 g/L); N: Urea (0 g/L–4 g/L); O: Potassium nitrate (0 g/L–1 g/L); P: Sodium acetate (0 g/L–5 g/L); Q: Magnesium sulphate (0 g/L–0.2 g/L); R: Manganese sulphate (0 g/L–0.04 g/L); S: Dipotassium hydrogen phosphate (0 g/L–2 g/L); and T: Tween 80 (0 mL/L–1 mL/L). Values are mean ± SEM, *n* = 3. Mean ± SEM within the same column that share a common superscript (a–i) are not significantly different (*p* > 0.05).

**Table 2:** Analysis of variance of Plackett–Burman Design for the effect assessments of medium components on extracellular xylanase production by *P. pentosaceus* G4.

Source	Sum of Squares	df	Mean Square	F-Value	*p*-Value	
Model	1.40	15	0.0931	159.83	<0.0001	Significant
A—Glucose	0.2121	1	0.2121	364.30	<0.0001	
B—Almond shell	0.1905	1	0.1905	327.10	<0.0001	
C—Peanut shell	0.1870	1	0.1870	321.10	<0.0001	
D—Hazelnut shell	0.0130	1	0.0130	22.34	0.0091	
E—Pistachio shell	0.0738	1	0.0738	126.75	0.0004	
F—Walnut shell	0.0198	1	0.0198	34.05	0.0043	
G—Malt extract	0.0054	1	0.0054	9.22	0.0385	
H—Xylan	0.1903	1	0.1903	326.83	<0.0001	
J—Peptone	0.0389	1	0.0389	66.76	0.0012	
K—Yeast extract	0.2222	1	0.2222	381.70	<0.0001	
L—Meat extract	0.0372	1	0.0372	63.94	0.0013	
N—Urea	0.1384	1	0.1384	237.69	0.0001	
P—Sodium acetate	0.0288	1	0.0288	49.48	0.0022	
Q—Magnesium sulphate	0.0266	1	0.0266	45.62	0.0025	
S—Dipotassium hydrogen phosphate	0.0120	1	0.0120	20.63	0.0105	
Residual	0.0023	4	0.0006			
Cor Total	1.40	19				

Notes: R^2^: 0.9983; Adjusted R^2^: 0.9921; Predicted R^2^: 0.9584; and Adequate precision: 48.2251.

**Table 3:** Specific extracellular xylanase activity, cell population, lactic acid concentration, utilised glucose concentration, initial and final pH corresponding to the Central Composite Design experimental runs.

Experimental Run	Specific Extracellular Xylanase Activity (U/mg)		Cell Population (Log CFU/mL)		Lactic Acid (g/mL)	Utilised Sugar (g/L)	Initial pH	Final pH
	Experimental	Predicted *	Experimental	Predicted *	Experimental	Experimental	Experimental	Experimental
1	0.1888 ^no^ ± 0.0456	−0.1308	7.8030 ^klm^ ± 0.0906	7.73844	11.0105 ^j^ ± 0.0101	4.6196 ^m^ ± 0.0984	6.21	4.85
2	0.5277 ^ijklmno^ ± 0.0655	0.7697	7.8667 ^ijklm^ ± 0.1191	7.90031	13.1940 ^ghij^ ± 0.0253	6.6145 ^ijklm^ ± 0.1873	6.23	4.66
3	0.2773 ^lmno^ ± 0.0644	0.4908	7.8127 ^jklm^ ± 0.0737	7.84304	11.2079 ^j^ ± 0.0209	5.0546 ^lm^ ± 0.1171	6.25	4.77
4	1.6239 ^bcdefghij^ ± 0.0951	1.0844	8.0794 ^bcdefgh^ ± 0.0919	7.96835	14.7503 ^abcdefghi^ ± 0.4851	8.9243 ^cdefghij^ ± 0.1050	6.22	4.51
5	1.8737 ^abcdefgh^ ± 0.0439	1.6952	8.0961 ^bcdefgh^ ± 0.0653	8.05298	15.0929 ^abcdefghi^ ± 0.0419	9.3443 ^cdefghi^ ± 0.1281	6.26	4.45
6	2.6942 ^abc^ ± 0.0751	2.5828	8.2407 ^abcd^ ± 0.0668	8.21065	16.8351 ^abc^ ± 0.0253	11.5041 ^abc^ ± 1.4970	6.21	4.30
7	1.8624 ^abcdefgh^ ± 0.0551	1.4892	8.0918 ^bcdefgh^ ± 0.0730	8.01477	15.0830 ^abcdefghi^ ± 0.0041	9.3892 ^cdefghi^ ± 0.0794	6.21	4.45
8	1.9088 ^abcdefgh^ ± 0.1221	2.0696	8.0948 ^bcdefgh^ ± 0.0201	8.13588	15.5981 ^abcdefgh^ ± 0.0290	9.4492 ^cdefghi^ ± 0.0937	6.28	4.44
9	0.5541 ^ijklmno^ ± 0.0778	0.5354	7.8934 ^hijklm^ ± 0.1061	7.87604	13.3914 ^efghij^ ± 0.0116	6.9894 ^ghijklm^ ± 0.1333	6.4	4.66
10	1.4579 ^efghijkl^ ± 0.0715	1.3004	8.0546 ^cdefghi^ ± 0.0527	7.9939	14.5354 ^abcdefghi^ ± 0.0838	8.5493 ^cdefghijk^ ± 0.0520	6.4	4.56
11	1.4135 ^efghijkl^ ± 0.0661	1.3013	8.0212 ^efghi^ ± 0.1247	8.0169	14.3902 ^bcdefghi^ ± 0.0402	8.3993 ^cdefghijkl^ ± 0.1171	6.42	4.57
12	1.7159 ^bcdefghi^ ± 0.0225	1.7591	8.0833 ^bcdefgh^ ± 0.0757	8.0982	14.9129 ^abcdefghi^ ± 0.0363	9.0893 ^cdefghij^ ± 0.2810	6.44	4.47
13	1.2931 ^efghijklmn^ ± 0.0665	1.0425	7.9972 ^efghijk^ ± 0.0725	7.9646	14.2102 ^cdefghi^ ± 0.0307	8.2793 ^cdefghijkl^ ± 0.0260	6.41	4.57
14	1.8652 ^abcdefgh^ ± 0.0233	1.7945	8.0835 ^bcdefgh^ ± 0.0464	8.0782	15.0174 ^abcdefghi^ ± 0.0101	9.4942 ^bcdefghi^ ± 0.2637	6.42	4.47
15	0.5771 ^ijklmno^ ± 0.0400	0.9806	7.9263 ^ghijklm^ ± 0.0397	7.9627	13.2346 ^fghij^ ± 0.0323	6.7945 ^hijklm^ ± 0.0937	6.41	4.66
16	1.6574 ^bcdefghij^ ± 0.0700	1.42542	8.0794 ^bcdefgh^ ± 0.0934	8.0397	14.8548 ^abcdefghi^ ± 0.0058	9.3743 ^cdefghi^ ± 0.1308	6.43	4.47
17	0.6448 ^ijklmno^ ± 0.0069	0.5471	7.9284 ^ghijklm^ ± 0.0243	7.8885	13.6005 ^efghij^ ± 0.0209	8.0244 ^defghijklm^ ± 0.1308	6.28	4.60
18	1.4518 ^efghijkl^ ± 0.0958	1.2178	8.0101 ^efghij^ ± 0.0744	7.9987	14.4483 ^bcdefghi^ ± 0.1057	8.4143 ^cdefghijkl^ ± 0.0937	6.21	4.56
19	0.8933 ^hijklmno^ ± 0.0420	0.8879	7.9512 ^fghijklm^ ± 0.0697	7.9305	13.7398 ^defghij^ ± 0.1424	8.0544 ^cdefghijklm^ ± 0.1580	6.29	4.58
20	1.0771 ^fghijklmno^ ± 0.0838	1.2514	7.9894 ^efghijkl^ ± 0.0521	8.0041	14.1696 ^cdefghi^ ± 0.0154	8.1443 ^cdefghijkl^ ± 0.1873	6.22	4.58
21	1.5047 ^cdefghijk^ ± 0.0476	1.8441	8.0687 ^bcdefghi^ ± 0.0746	8.1307	14.7677 ^abcdefghi^ ± 0.1108	8.9693 ^cdefghij^ ± 0.0794	6.27	4.53
22	2.9243 ^a^ ± 0.0919	2.5016	8.3531 ^a^ ± 0.1033	8.2366	17.3171 ^a^ ± 0.0201	13.1090 ^a^ ± 0.9382	6.23	4.21
23	1.4889 ^defghijk^ ± 0.0116	1.3571	8.0390 ^defghi^ ± 0.0447	8.0299	14.5470 ^abcdefghi^ ± 0.1220	8.6093 ^cdefghijk^ ± 0.1950	6.22	4.56
24	1.5597 ^cdefghijk^ ± 0.0219	1.7075	8.0665 ^bcdefghi^ ± 0.1074	8.0992	14.6516 ^abcdefghi^ ± 0.0363	8.8943 ^cdefghij^ ± 0.1670	6.21	4.55
25	0.8473 ^hijklmno^ ± 0.0317	0.9080	7.9674 ^fghijklm^ ± 0.0685	7.9561	13.5482 ^efghij^ ± 0.0058	7.4439 ^efghijklm^ ± 0.1043	6.43	4.64
26	1.4436 ^efghijkl^ ± 0.0991	1.4430	8.0187 ^efghi^ ± 0.0379	8.0222	14.5122 ^abcdefghi^ ± 0.0266	8.4743 ^cdefghijkl^ ± 0.1281	6.41	4.56
27	1.4097 ^efghijklm^ ± 0.0324	1.3928	8.0269 ^efghi^ ± 0.0413	8.0343	14.3438 ^bcdefghi^ ± 0.0209	8.3483 ^cdefghijkl^ ± 0.0323	6.42	4.57
28	1.4742 ^efghijkl^ ± 0.0663	1.6207	8.0526 ^cdefghi^ ± 0.0898	8.0639	14.6748 ^abcdefghi^ ± 0.1108	8.8643 ^cdefghij^ ± 0.2505	6.4	4.53
29	0.9333 ^ghijklmno^ ± 0.0631	0.8859	7.9867 ^efghijkl^ ± 0.0522	7.9723	13.7224 ^defghij^ ± 0.0253	8.0799 ^cdefghijklm^ ± 0.7327	6.44	4.58
30	1.1946 ^efghijklmno^ ± 0.0547	1.4078	7.9935 ^efghijk^ ± 0.1328	8.0342	14.2044 ^cdefghi^ ± 0.0154	8.3093 ^cdefghijkl^ ± 0.2602	6.43	4.58
31	0.6038 ^ijklmno^ ± 0.0120	0.54299	7.9212 ^ghijklm^ ± 0.0323	7.9077	13.5017 ^efghij^ ± 0.0266	7.0899 ^fghijklm^ ± 0.0437	6.41	4.65
32	0.7716 ^hijklmno^ ± 0.0313	0.75780	7.9324 ^ghijklm^ ± 0.0556	7.9330	13.5947 ^efghij^ ± 0.0154	8.0244 ^defghijklm^ ± 0.1587	6.43	4.59
33	0.0000 ^o^ ± 0.0000	0.09803	7.7740 ^m^ ± 0.1687	7.8150	6.2253 ^k^ ± 0.1192	0.0000 ^n^ ± 0.0000	6.37	5.36
34	1.3018 ^efghijklmn^ ± 0.0442	1.42451	8.0094 ^efghij^ ± 0.0462	8.0377	14.2218 ^cdefghi^ ± 0.0768	8.2943 ^cdefghijkl^ ± 0.0541	6.31	4.57
35	0.5331 ^ijklmno^ ± 0.0906	0.82385	7.8749 ^ijklm^ ± 0.0681	7.9561	13.2462 ^fghij^ ± 0.1018	6.8994 ^hijklm^ ± 0.1050	6.31	4.68
36	0.8602 ^hijklmno^ ± 0.0678	0.7901	7.9722 ^efghijklm^ ± 0.0231	7.9603	13.6760 ^efghij^ ± 0.0612	8.0844 ^cdefghijkl^ ± 0.1522	6.35	4.58
37	0.2138 ^mno^ ± 0.0929	0.3228	7.7893 ^lm^ ± 0.0812	7.8361	12.3461 ^ij^ ± 0.0058	5.2796 ^klm^ ± 0.0150	6.32	4.77
38	1.3565 ^efghijklmn^ ± 0.0600	1.4682	8.0321 ^efghi^ ± 0.0947	8.0546	13.8211 ^defghij^ ± 0.0307	8.1593 ^cdefghijkl^ ± 0.0600	6.38	4.58
39	0.3886 ^klmno^ ± 0.0556	0.7137	7.8075 ^jklm^ ± 0.0280	7.8894	12.6829 ^ij^ ± 0.0402	5.6545 ^jklm^ ± 0.0150	6.11	4.73
40	0.4811 ^jklmno^ ± 0.0642	0.3766	7.8680 ^ijklm^ ± 0.0557	7.8554	12.8571 ^hij^ ± 0.0201	6.5995 ^ijklm^ ± 0.0912	6.49	4.68
41	2.2082 ^abcdef^ ± 0.0705	2.6027	8.1059 ^bcdefg^ ± 0.1303	8.1965	15.6794 ^abcdefgh^ ± 0.0302	9.7192 ^abcdefghi^ ± 0.0260	6.32	4.43
42	2.7889 ^ab^ ± 0.0787	2.6151	8.2694 ^ab^ ± 0.0686	8.2481	17.1719 ^ab^ ± 0.0058	12.9440 ^ab^ ± 0.0750	6.3	4.22
43	2.2604 ^abcdef^ ± 0.0929	2.3373	8.1396 ^bcdef^ ± 0.1079	8.1515	16.0221 ^abcdefg^ ± 0.0154	10.4992 ^abcdef^ ± 0.1200	6.3	4.43
44	2.2200 ^abcdef^ ± 0.0986	2.3373	8.1022 ^bcdefg^ ± 0.0786	8.1515	16.0046 ^abcdefg^ ± 0.1366	10.1392 ^abcdefgh^ ± 0.0541	6.3	4.41
45	2.3411 ^abcde^ ± 0.1555	2.3373	8.1743 ^abcde^ ± 0.0568	8.1515	16.5621 ^abcd^ ± 0.0253	10.8441 ^abcde^ ± 0.1800	6.3	4.37
46	2.3140 ^abcde^ ± 0.1454	2.3373	8.1436 ^bcdef^ ± 0.0500	8.1515	16.1847 ^abcde^ ± 0.0101	10.5892 ^abcde^ ± 0.1050	6.3	4.38
47	2.1264 ^abcdefg^ ± 0.0970	2.3373	8.1004 ^bcdefg^ ± 0.0371	8.1515	15.8885 ^abcdefg^ ± 0.0266	10.1242 ^abcdefgh^ ± 0.0937	6.3	4.43
48	2.6822 ^abcd^ ± 0.0323	2.3373	8.2436 ^abc^ ± 0.0510	8.1515	15.7607 ^abcdefg^ ± 0.0116	9.9592 ^abcdefghi^ ± 0.2163	6.3	4.44
49	2.2831 ^abcde^ ± 0.1130	2.3373	8.1186 ^bcdefg^ ± 0.0257	8.1515	16.0859 ^abcdef^ ± 0.2125	10.3792 ^abcdefg^ ± 0.2116	6.3	4.41
50	2.3265 ^abcde^ ± 0.0481	2.3373	8.1442 ^bcdef^ ± 0.0614	8.1515	16.5854 ^abcd^ ± 0.0402	11.1141 ^abcd^ ± 0.1819	6.3	4.31

Notes: Values are mean ± standard error of the mean (SEM), *n* = 3. Mean ± SEM within the same column that share similar superscript letters are not significantly different (*p* > 0.05). * Predicted extracellular xylanase production and cell population were calculated based on Equations (2) and (3), respectively.

**Table 4:** ANOVA of the CCD suggested a regression model of optimised formulated medium and extracellular xylanase activity of *P. pentosaceus* G4.

Regression Model	Sequential *p*-Value	Lack of Fit *p*-Value	Adjusted R^2^	Predicted R^2^	
Linear	0.0517	0.0001	0.1254	0.0078	
2FI	0.2764	0.0001	0.1790	0.1708	
Quadratic	<0.0001	0.0502	0.8714	0.7323	Suggested
Cubic	0.6845	0.0202	0.8545	−0.6719	Aliased

**Table 5:** ANOVA for the quadratic polynomial model of the optimised formulated medium and extracellular xylanase production of *P. pentosaceus* G4.

Source	Sum of Squares	df	Mean Square	F-Value	*p*-Value	
Model	26.25	20	1.31	17.60	<0.0001	Significant
A—Glucose	3.37	1	3.37	45.15	<0.0001	Significant
B—Almond shell	0.0022	1	0.0022	0.0291	0.8658	
C—Peanut shell	2.51	1	2.51	33.67	<0.0001	Significant
D—Urea	0.2175	1	0.2175	2.92	0.0984	
E—Magnesium sulphate	0.0003	1	0.0003	0.0039	0.9504	
AB	0.1886	1	0.1886	2.53	0.1226	
AC	0.0003	1	0.0003	0.0046	0.9465	
AD	0.0368	1	0.0368	0.4933	0.4881	
AE	0.1058	1	0.1058	1.42	0.2434	
BC	1.37	1	1.37	18.37	0.0002	Significant
BD	0.0415	1	0.0415	0.5566	0.4616	
BE	0.1579	1	0.1579	2.12	0.1565	
CD	3.48	1	3.48	46.65	<0.0001	Significant
CE	0.5601	1	0.5601	7.51	0.0104	Significant
DE	0.1866	1	0.1866	2.50	0.1245	
A^2^	4.31	1	4.31	57.82	<0.0001	Significant
B^2^	4.07	1	4.07	54.52	<0.0001	Significant
C^2^	3.61	1	3.61	48.39	<0.0001	Significant
D^2^	5.58	1	5.58	74.76	<0.0001	Significant
E^2^	0.1281	1	0.1281	1.72	0.2004	
Residual	2.16	29	0.0746			
Lack of Fit	1.98	22	0.0900	3.42	0.0502	Not significant
Pure Error	0.1841	7	0.0263			
Cor Total	28.42	49				

Notes: R^2^: 0.9239; Adjusted R^2^: 0.8714; Pred R^2^: 0.7323; and Adequate precision: 15.5135.

**Table 6:** Comparison of media costs for extracellular xylanase production by *P. pentosaceus* G4.

Media	Specific Extracellular Xylanase Activity (U/mg)	Medium Composition (g/L)	Medium Cost (USD/L)		Increase Specific Extracellular Xylanase Activity (Fold)	Medium Cost Reduction (Fold)
			Composition	Total		
MRS	0.8809	-		11.11	Baseline (1.00)	Baseline (1.00)
Optimised Medium	2.7646	Glucose	0.96	1.391	3.13	7.99
		Almond Shell	Free			
		Peanut Shell	Free			
		Urea	0.40			
		Magnesium Sulphate	0.031			

**Table 7:** ANOVA of regression models of optimised formulated medium and cell population of *P. pentosaceus* G4.

Source	Sequential *p*-Value	Lack of Fit *p*-Value	Adjusted R^2^	Predicted R^2^	
Linear	0.0255	0.0054	0.1594	0.0271	
2FI	0.1688	0.0070	0.2509	0.2003	
Quadratic	<0.0001	0.2045	0.7790	0.5415	Suggested
Cubic	0.8328	0.0759	0.7210	−3.2719	Aliased

**Table 8:** ANOVA for the quadratic model of the optimised formulated medium and cell population of *P. pentosaceus* G4.

Source	Sum of Squares	df	Mean Square	F-Value	*p*-Value	
Model	0.6864	20	0.0343	9.64	<0.0001	Significant
A—Glucose	0.0949	1	0.0949	26.65	<0.0001	Significant
B—Almond shell	0.0000	1	0.0000	0.0092	0.9241	
C—Peanut shell	0.0914	1	0.0914	25.66	<0.0001	Significant
D—Urea	0.0022	1	0.0022	0.6206	0.4372	
E—Magnesium sulphate	0.0051	1	0.0051	1.44	0.2406	
AB	0.0027	1	0.0027	0.7507	0.3934	
AC	0.0000	1	0.0000	0.0100	0.9212	
AD	0.0039	1	0.0039	1.09	0.3056	
AE	0.0054	1	0.0054	1.50	0.2300	
BC	0.0408	1	0.0408	11.45	0.0021	Significant
BD	0.0026	1	0.0026	0.7395	0.3969	
BE	0.0078	1	0.0078	2.20	0.1485	
CD	0.1021	1	0.1021	28.67	<0.0001	Significant
CE	0.0105	1	0.0105	2.94	0.0969	
DE	0.0098	1	0.0098	2.76	0.1075	
A^2^	0.0880	1	0.0880	24.71	<0.0001	Significant
B^2^	0.0649	1	0.0649	18.21	0.0002	Significant
C^2^	0.0738	1	0.0738	20.72	<0.0001	Significant
D^2^	0.1352	1	0.1352	37.98	<0.0001	Significant
E^2^	0.0087	1	0.0087	2.45	0.1287	
Residual	0.1033	29	0.0036			
Lack of Fit	0.0882	22	0.0040	1.85	0.2045	Not significant
Pure Error	0.0151	7	0.0022			
Cor Total	0.7897	49				

Notes: R^2^: 0.8692; Adjusted R^2^: 0.7790; Predicted R^2^: 0.5415; and Adequate precision: 13.1801.

**Table 9:** Coded and actual values of each medium component of Plackett–Burman Design for extracellular xylanase production of *P. pentosaceus* G4.

No.	Medium Component	Symbol Code	Concentration Unit	Coded Values	
				−1	+1
1.	Glucose	A	g/L	0	20
2.	Almond shell	B	g/L	0	20
3.	Peanut shell	C	g/L	0	20
4.	Hazelnut shell	D	g/L	0	20
5.	Pistachio shell	E	g/L	0	20
6.	Walnut shell	F	g/L	0	20
7	Malt extract	G	g/L	0	5
8.	Xylan	H	g/L	0	20
9.	Peptone	J	g/L	0	10
10.	Yeast extract	K	g/L	0	4
11.	Meat extract	L	g/L	0	8
12.	Ammonium citrate	M	g/L	0	2
13.	Urea	N	g/L	0	4
14.	Potassium nitrate	O	g/L	0	1
15.	Sodium acetate	P	g/L	0	5
16.	Magnesium sulphate	Q	g/L	0	0.2
18.	Manganese sulphate	R	g/L	0	0.04
18.	Dipotassium hydrogen phosphate	S	g/L	0	2
19.	Tween 80	T	ml/L	0	1

**Table 10:** Plackett–Burman Design matrices of the experimental runs for extracellular xylanase production of *P. pentosaceus* G4.

Experimental Run	A	B	C	D	E	F	G	H	J	K	L	M	N	O	P	Q	R	S	T
1	1	1	−1	−1	1	1	1	1	−1	1	−1	1	−1	−1	−1	−1	1	1	−1
2	−1	1	1	−1	−1	1	1	1	1	−1	1	−1	1	−1	−1	−1	−1	1	1
3	1	−1	1	1	−1	−1	1	1	1	1	−1	1	−1	1	−1	−1	−1	−1	1
4	1	1	−1	1	1	−1	−1	1	1	1	1	−1	1	−1	1	−1	−1	−1	−1
5	−1	1	1	−1	1	1	−1	−1	1	1	1	1	−1	1	−1	1	−1	−1	−1
6	−1	−1	1	1	−1	1	1	−1	−1	1	1	1	1	−1	1	−1	1	−1	−1
7	−1	−1	−1	1	1	−1	1	1	−1	−1	1	1	1	1	−1	1	−1	1	−1
8	−1	−1	−1	−1	1	1	−1	1	1	−1	−1	1	1	1	1	−1	1	−1	1
9	1	−1	−1	−1	−1	1	1	−1	1	1	−1	−1	1	1	1	1	−1	1	−1
10	−1	1	−1	−1	−1	−1	1	1	−1	1	1	−1	−1	1	1	1	1	−1	1
11	1	−1	1	−1	−1	−1	−1	1	1	−1	1	1	−1	−1	1	1	1	1	−1
12	−1	1	−1	1	−1	−1	−1	−1	1	1	−1	1	1	−1	−1	1	1	1	1
13	1	−1	1	−1	1	−1	−1	−1	−1	1	1	−1	1	1	−1	−1	1	1	1
14	1	1	−1	1	−1	1	−1	−1	−1	−1	1	1	−1	1	1	−1	−1	1	1
15	1	1	1	−1	1	−1	1	−1	−1	−1	−1	1	1	−1	1	1	−1	−1	1
16	1	1	1	1	−1	1	−1	1	−1	−1	−1	−1	1	1	−1	1	1	−1	−1
17	−1	1	1	1	1	−1	1	−1	1	−1	−1	−1	−1	1	1	−1	1	1	−1
18	−1	−1	1	1	1	1	−1	1	−1	1	−1	−1	−1	−1	1	1	−1	1	1
19	1	−1	−1	1	1	1	1	−1	1	−1	1	−1	−1	−1	−1	1	1	−1	1
20	−1	−1	−1	−1	−1	−1	−1	−1	−1	−1	−1	−1	−1	−1	−1	−1	−1	−1	−1

**Table 11:** Coded and actual values of the medium component of the Central Composite Design for extracellular xylanase production of *P. pentosaceus* G4.

Medium Components	Coded Symbol	Coded Values				
		−α	−1	0	+1	+α
Glucose	A	0	10	20	30	40
Almond shell	B	0	10	20	30	40
Peanut shell	C	0	10	20	30	40
Urea	D	0	2	4	6	8
Magnesium sulphate	E	0	0.1	0.2	0.3	0.4

**Table 12:** Central Composite Design matrices for extracellular xylanase production of *P. pentosaceus* G4.

Experimental Run	A	B	C	D	E
1	−1	−1	−1	−1	−1
2	1	−1	−1	−1	−1
3	−1	1	−1	−1	−1
4	1	1	−1	−1	−1
5	−1	−1	1	−1	−1
6	1	−1	1	−1	−1
7	−1	1	1	−1	−1
8	1	1	1	−1	−1
9	−1	−1	−1	1	−1
10	1	−1	−1	1	−1
11	−1	1	−1	1	−1
12	1	1	−1	1	−1
13	−1	−1	1	1	−1
14	1	−1	1	1	−1
15	−1	1	1	1	−1
16	1	1	1	1	−1
17	−1	−1	−1	−1	1
18	1	−1	−1	−1	1
19	−1	1	−1	−1	1
20	1	1	−1	−1	1
21	−1	−1	1	−1	1
22	1	−1	1	−1	1
23	−1	1	1	−1	1
24	1	1	1	−1	1
25	−1	−1	−1	1	1
26	1	−1	−1	1	1
27	−1	1	−1	1	1
28	1	1	−1	1	1
29	−1	−1	1	1	1
30	1	−1	1	1	1
31	−1	1	1	1	1
32	1	1	1	1	1
33	−α	0	0	0	0
34	+α	0	0	0	0
35	0	−α	0	0	0
36	0	+α	0	0	0
37	0	0	−α	0	0
38	0	0	+α	0	0
39	0	0	0	−α	0
40	0	0	0	+α	0
41	0	0	0	0	−α
42	0	0	0	0	+α
43	0	0	0	0	0
44	0	0	0	0	0
45	0	0	0	0	0
46	0	0	0	0	0
47	0	0	0	0	0
48	0	0	0	0	0
49	0	0	0	0	0
50	0	0	0	0	0

## Data Availability

The original contributions presented in this study are included in the article. Further inquiries can be directed to the corresponding authors.

## References

[1] Saavedra-Bouza A., Escuder-Rodríguez J.-J., deCastro M.-E., Becerra M., González-Siso M.-I. (2023). Xylanases from thermophilic archaea: A hidden treasure. Curr. Res. Biotechnol..

[2] Mendonça M., Barroca M., Collins T. (2023). Endo-1,4-β-xylanase-containing glycoside hydrolase families: Characteristics, singularities and similarities. Biotechnol. Adv..

[3] Devi S., Dwivedi D., Bhatt A.K. (2022). Utilization of agroresidues for the production of xylanase by *Bacillus safensis* XPS7 and optimization of production parameters. Fermentation.

[4] Chakdar H., Kumar M., Pandiyan K., Singh A., Nanjappan K., Kashyap P.L., Srivastava A.K. (2016). Bacterial xylanases: Biology to biotechnology. 3 Biotech.

[5] Shrestha S., Chio C., Khatiwada J.R., Kognou A.L.M., Qin W. (2022). Formulation of the agro-waste mixture for multi-enzyme (pectinase, xylanase, and cellulase) production by mixture design method exploiting *Streptomyces* sp.. Bioresour. Technol. Rep..

[6] Nabais J.M.V., Laginhas C.E.C., Carrott P.J.M., Ribeiro Carrott M.M.L. (2011). Production of activated carbons from almond shell. Fuel Process. Technol..

[7] Di Michele A., Pagano C., Allegrini A., Blasi F., Cossignani L., Raimo E.D., Faieta M., Oliva E., Pittia P., Primavilla S. (2021). Hazelnut shells as source of active ingredients: Extracts preparation and characterization. Molecules.

[8] Pączkowski P., Puszka A., Gawdzik B. (2021). Effect of eco-friendly peanut shell powder on the chemical resistance, physical, thermal, and thermomechanical properties of unsaturated polyester resin Composites. Polymers.

[9] Bilal M., Iqbal H.M.N. (2020). Recent advancements in the life cycle analysis of lignocellulosic biomass. Curr. Sustain./Renew. Energy Rep..

[10] Dwivedi P., Vivekanand V., Ganguly R., Singh R.P. (2009). *Parthenium* sp. as a plant biomass for the production of alkalitolerant xylanase from mutant *Penicillium oxalicum* SAUE-3.510 in submerged fermentation. Biomass Bioenergy.

[11] Terrone C.C., Freitas C.D., Terrasan C.R.F., Almeida A.F.D., Carmona E.C. (2018). Agroindustrial biomass for xylanase production by *Penicillium chrysogenum*: Purification, biochemical properties and hydrolysis of hemicelluloses. Electron. J. Biotechnol..

[12] Sadaf A., Khare S.K. (2014). Production of *Sporotrichum thermophile* xylanase by solid state fermentation utilizing deoiled Jatropha curcas seed cake and its application in xylooligosachharide synthesis. Bioresour. Technol..

[13] Kim J., Kim Y.-M., Lebaka V.R., Wee Y.-J. (2022). Lactic acid for green chemical industry: Recent advances in and future prospects for production technology, recovery, and applications. Fermentation.

[14] Bhardwaj N., Kumar B., Verma P. (2019). A detailed overview of xylanases: An emerging biomolecule for current and future prospective. Bioresour. Bioprocess..

[15] Allikian K., Edgar R., Syed R., Zhang S., Berenjian A. (2019). Fundamentals of Fermentation Media. Essentials in Fermentation Technology.

[16] Panda B.P., Ali M., Javed S. (2007). Fermentation process optimization. Res. J. Microbiol..

[17] Lim Y.H., Foo H.L., Loh T.C., Mohamad R., Abdul Rahim R. (2020). Rapid evaluation and optimization of medium components governing tryptophan production by *Pediococcus acidilactici* TP-6 isolated from Malaysian food via statistical approaches. Molecules.

[18] Limkar M.B., Pawar S.V., Rathod V.K. (2019). Statistical optimization of xylanase and alkaline protease co-production by *Bacillus* spp. using Box-Behnken Design under submerged fermentation using wheat bran as a substrate. Biocatal. Agric. Biotechnol..

[19] Singh V., Haque S., Niwas R., Srivastava A., Pasupuleti M., Tripathi C.K.M. (2017). Strategies for fermentation medium optimization: An in-depth review. Front. Microbiol..

[20] Topakas E., Panagiotou G., Christakopoulos P., Yang S.-T., El-Enshasy H.A., Thongchul N. (2013). Xylanases: Characteristics, sources, production, and applications. Bioprocessing Technologies in Biorefinery for Sustainable Production of Fuels, Chemicals, and Polymers.

[21] Jacyna J., Kordalewska M., Markuszewski M.J. (2019). Design of experiments in metabolomics-related studies: An overview. J. Pharm. Biomed. Anal..

[22] Dhaver P., Pletschke B., Sithole B., Govinden R. (2022). Optimization, purification, and characterization of xylanase production by a newly isolated *Trichoderma harzianum* strain by a two-step statistical experimental design strategy. Sci. Rep..

[23] Walia A., Guleria S., Mehta P., Chauhan A., Parkash J. (2017). Microbial xylanases and their industrial application in pulp and paper biobleaching: A review. 3 Biotech.

[24] Coman G., Bahrim G. (2011). Optimization of xylanase production by *Streptomyces* sp. P12-137 using response surface methodology and central composite design. Ann. Microbiol..

[25] Bibra M., Kunreddy V.R., Sani R.K. (2018). Thermostable xylanase production by *Geobacillus* sp. strain DUSELR13, and its application in ethanol production with lignocellulosic biomass. Microorganisms.

[26] Thite V.S., Nerurkar A.S., Baxi N.N. (2020). Optimization of concurrent production of xylanolytic and pectinolytic enzymes by *Bacillus safensis* M35 and *Bacillus altitudinis* J208 using agro-industrial biomass through response surface methodology. Sci. Rep..

[27] Fernandes de Souza H., Aguiar Borges L., Dédalo Di Próspero Gonçalves V., Vitor dos Santos J., Sousa Bessa M., Fronja Carosia M., Vieira de Carvalho M., Viana Brandi I., Setsuko Kamimura E. (2022). Recent advances in the application of xylanases in the food industry and production by actinobacteria: A review. Food Res. Int..

[28] Pariza M.W., Johnson E.A. (2001). Evaluating the safety of microbial enzyme preparations used in food processing: Update for a new century. Regul. Toxicol. Pharmacol..

[29] Lee F.H., Wan S.Y., Foo H.L., Loh T.C., Mohamad R., Abdul Rahim R., Idrus Z. (2019). Comparative study of extracellular proteolytic, cellulolytic, and hemicellulolytic enzyme activities and biotransformation of palm kernel cake biomass by lactic acid bacteria isolated from Malaysian foods. Int. J. Mol. Sci..

[30] Zabidi N.A.M., Foo H.L., Loh T.C., Mohamad R., Abdul Rahim R. (2020). Enhancement of versatile extracellular cellulolytic and hemicellulolytic enzyme productions by *Lactobacillus plantarum* RI 11 isolated from Malaysian food using renewable natural polymers. Molecules.

[31] Li Y., Liu Z., Zhao H., Xu Y., Cui F. (2007). Statistical optimization of xylanase production from new isolated *Penicillium oxalicum* ZH-30 in submerged fermentation. Biochem. Eng. J..

[32] Bedade D., Berezina O., Singhal R., Deska J., Shamekh S. (2017). Extracellular xylanase production from a new xylanase producer *Tuber maculatum* mycelium under submerged fermentation and its characterization. Biocatal. Agric. Biotechnol..

[33] Nagar S., Gupta V.K., Kumar D., Kumar L., Kuhad R.C. (2010). Production and optimization of cellulase-free, alkali-stable xylanase by *Bacillus pumilus* SV-85S in submerged fermentation. J. Ind. Microbiol. Biotechnol..

[34] Ikram-ul-Haq M.M.J., Khan T.S. (2006). An innovative approach for hyperproduction of cellulolytic and hemicellulolytic enzymes by consortium of *Aspergillus niger* MSK-7 and *Trichoderma viride* MSK-10. Afr. J. Biotechnol..

[35] Jecu L. (2000). Solid state fermentation of agricultural wastes for endoglucanase production. Ind. Crops Prod..

[36] Gowdhaman D., Manaswini V.S., Jayanthi V., Dhanasri M., Jeyalakshmi G., Gunasekar V., Sugumaran K.R., Ponnusami V. (2014). Xylanase production from *Bacillus aerophilus* KGJ2 and its application in xylooligosaccharides preparation. Int. J. Biol. Macromol..

[37] Velkova Z., Gocheva V., Kostov G., Atev A. (2007). Optimization of nutritive media composition for hylanase production be *Aspergillus awamori*. Bulg. J. Agric. Sci..

[38] Lama L., Calandrelli V., Gambacorta A., Nicolaus B. (2004). Purification and characterization of thermostable xylanase and β-xylosidase by the thermophilic bacterium *Bacillus thermantarcticus*. Res. Microbiol..

[39] Mmango-Kaseke Z., Okaiyeto K., Nwodo U.U., Mabinya L.V., Okoh A.I. (2016). Optimization of cellulase and xylanase production by *Micrococcus* species under submerged fermentation. Sustainability.

[40] Duarte M.C.T., Portugal E.P., Ponezi A.N., Bim M.A., Tagliari C.V., Franco T.T. (1999). Production and purification of alkaline xylanases. Bioresour. Technol..

[41] Bhalla A., Bischoff K.M., Sani R.K. (2015). Highly thermostable xylanase production from a thermophilic *Geobacillus* sp. strain WSUCF1 utilizing lignocellulosic biomass. Front. Bioeng. Biotechnol..

[42] Blanco A., Vidal T., Colom J.F., Pastor F. (1995). Purification and properties of xylanase A from alkali-tolerant *Bacillus* sp. strain BP-23. Appl. Environ. Microbiol..

[43] Tseng M.-J., Yap M.-N., Ratanakhanokchai K., Kyu K.L., Chen S.-T. (2002). Purification and characterization of two cellulase free xylanases from an alkaliphilic *Bacillus firmus*. Enzym. Microb. Technol..

[44] Poosarla V.G., Chandra T. (2014). Purification and characterization of novel halo-acid-alkali-thermo-stable xylanase from *Gracilibacillus* sp. TSCPVG. Appl. Biochem. Biotechnol..

[45] Giridhar P.V., Chandra T.S. (2010). Production of novel halo-alkali-thermo-stable xylanase by a newly isolated moderately halophilic and alkali-tolerant *Gracilibacillus* sp. TSCPVG. Process Biochem..

[46] Boucherba N., Gagaoua M., Copinet E., Bettache A., Duchiron F., Benallaoua S. (2014). Purification and characterization of the xylanase produced by *Jonesia denitrificans* BN-13. Appl. Biochem. Biotechnol..

[47] Dahlberg L., Holst O., Kristjansson J.K. (1993). Thermostable xylanolytic enzymes from *Rhodothermus marinus* grown on xylan. Appl. Microbiol. Biotechnol..

[48] Xin F., He J. (2013). Characterization of a thermostable xylanase from a newly isolated *Kluyvera* species and its application for biobutanol production. Bioresour. Technol..

[49] Dheeran P., Nandhagopal N., Kumar S., Jaiswal Y.K., Adhikari D.K. (2012). A novel thermostable xylanase of *Paenibacillus macerans* IIPSP3 isolated from the termite gut. J. Ind. Microbiol. Biotechnol..

[50] Seyis I., Aksoz N. (2005). Effect of carbon and nitrogen sources on xylanase production by *Trichoderma harzianum* 1073 D3. Int. Biodeterior. Biodegrad..

[51] Pasalari A., Homaei A. (2022). Isolation and molecular identification of xylanase-producing bacteria from *Ulva flexuosa* of the Persian Gulf. Processes.

[52] Ravanal M.-C., Rosa L., Polanco R., Eyzaguirre J., Espinosa Y., Levicán G., Chávez R., Vaca I. (2012). Glucose-induced production of a *Penicillium purpurogenum* xylanase by *Aspergillus nidulans*. Mycoscience.

[53] Knob A., Fortkamp D., Prolo T., Izidoro S.C., Almeida J.M. (2014). Agro-residues as alternative for xylanase production by filamentous fungi. BioResources.

[54] Wang Y., Wu J., Lv M., Shao Z., Hungwe M., Wang J., Bai X., Xie J., Wang Y., Geng W. (2021). Metabolism characteristics of lactic acid bacteria and the expanding applications in food industry. Front. Bioeng. Biotechnol..

[55] Mendonça E.H.M., Avanci N.C., Romano L.H., Branco D.L., de Pádua A.X., Ward R.J., Baptista Neto Á.D., Lourenzoni M.R. (2020). Recombinant xylanase production by *Escherichia coli* using a non-induced expression system with different nutrient sources. Braz. J. Chem. Eng..

[56] Shi X., Xie J., Liao S., Wu T., Zhao L.-G., Ding G., Wang Z., Xiao W. (2017). High-level expression of recombinant thermostable β-glucosidase in *Escherichia coli* by regulating acetic acid. Bioresour. Technol..

[57] Singh R.D., Nadar C.G., Muir J., Arora A. (2019). Green and clean process to obtain low degree of polymerisation xylooligosaccharides from almond shell. J. Clean. Prod..

[58] Ebringerová A., Hromádková Z., Košt’álová Z., Sasinková V. (2008). Chemical valorization of agricultural by-products: Isolation and characterization of xylan-based antioxidants from almond shell biomass. BioResources.

[59] Queirós C.S.G.P., Cardoso S., Lourenço A., Ferreira J., Miranda I., Lourenço M.J.V., Pereira H. (2020). Characterization of walnut, almond, and pine nut shells regarding chemical composition and extract composition. Biomass Convers. Biorefinery.

[60] Cho C.H., Hatsu M., Takamizawa K. (2002). The production of D-xylose by enzymatic hydrolysis of agricultural wastes. Water Sci. Technol..

[61] Barbieri G.S., Bento H.B.S., de Oliveira F., Picheli F.P., Dias L.M., Masarin F., Santos-Ebinuma V.C. (2022). Xylanase production by *Talaromyces amestolkiae* valuing agroindustrial byproducts. BioTech.

[62] Do T.T., Quyen D.T., Dam T.H. (2012). Purification and characterization of an acid-stable and organic solvent-tolerant xylanase from *Aspergillus awamori* VTCC-F312. ScienceAsia.

[63] Yang C.-H., Yang S.-F., Liu W.-H. (2007). Production of xylooligosaccharides from xylans by extracellular xylanases from *Thermobifida fusca*. J. Agric. Food Chem..

[64] Raju G., Kumarappa S., Gaitonde V. (2012). Mechanical and physical characterization of agricultural waste reinforced polymer composites. J. Mater. Environ. Sci..

[65] Arumugam N., Biely P., Puchart V., Singh S., Pillai S. (2018). Structure of peanut shell xylan and its conversion to oligosaccharides. Process Biochem..

[66] Kamble R.D., Jadhav A.R. (2012). Isolation, purification, and characterization of xylanase produced by a new species of *Bacillus* in solid state fermentation. Int. J. Microbiol..

[67] Paul M., Nayak D.P., Thatoi H. (2020). Optimization of xylanase from *Pseudomonas mohnii* isolated from Simlipal Biosphere Reserve, Odisha, using response surface methodology. J. Genet. Eng. Biotechnol..

[68] Kumar A.R., Hegde S.S., Ganesh K.N., Khan M.I. (2003). Structural changes enhance the activity of Chainia xylanase in low urea concentrations. Biochim. Biophys. Acta (BBA)—Proteins Proteom..

[69] Yin T., Miao L.L., Guan F.F., Wang G.L., Peng Q., Li B.X., Guan G.H., Li Y. (2010). Optimized medium improves expression and secretion of extremely thermostable bacterial xylanase, XynB, in *Kluyveromyces lactis*. J. Microbiol. Biotechnol..

[70] Sá-Pereira P., Mesquita A., Duarte J.C., Barros M.R.A., Costa-Ferreira M. (2002). Rapid production of thermostable cellulase-free xylanase by a strain of *Bacillus subtilis* and its properties. Enzym. Microb. Technol..

[71] Marimuthu M., Sorimuthu A., Muruganantham S. (2019). Production and optimization of xylanase enzyme from *Bacillus subtilis* using agricultural wastes by solid state fermentation. Int. J. Pharm. Investig..

[72] Ellatif S.A., Abdel Razik E.S., AL-surhanee A.A., Al-Sarraj F., Daigham G.E., Mahfouz A.Y. (2022). Enhanced production, cloning, and expression of a xylanase gene from endophytic fungal strain *Trichoderma harzianum* kj831197.1: Unveiling the in vitro anti-fungal activity against phytopathogenic fungi. J. Fungi.

[73] Battan B., Sharma J., Kuhad R. (2006). High-level xylanase production by alkaliphilic *Bacillus pumilus* ASH under solid-state fermentation. World J. Microbiol. Biotechnol..

[74] Sanghi A., Garg N., Sharma J., Kuhar K., Kuhad R.C., Gupta V.K. (2008). Optimization of xylanase production using inexpensive agro-residues by alkalophilic *Bacillus subtilis* ASH in solid-state fermentation. World J. Microbiol. Biotechnol..

[75] Adhyaru D.N., Bhatt N.S., Modi H.A. (2014). Enhanced production of cellulase-free, thermo-alkali-solvent-stable xylanase from *Bacillus altitudinis* DHN8, its characterization and application in sorghum straw saccharification. Biocatal. Agric. Biotechnol..

[76] Palaniswamy M., Pradeep B.V., Sathya R., Angayarkanni J. (2008). Isolation, identification and screening of potential xylanolytic enzyme from litter degrading fungi. Afr. J. Biotechnol..

[77] Amezaga M.R., Booth I.R. (1999). Osmoprotection of *Escherichia coli* by peptone is mediated by the uptake and accumulation of free proline but not of proline-containing peptides. Appl. Environ. Microbiol..

[78] Davami F., Eghbalpour F., Nematollahi L., Barkhordari F., Mahboudi F. (2015). Effects of peptone supplementation in different culture media on growth, metabolic pathway and productivity of CHO DG44 Cells; a new insight into amino acid profiles. Iran. Biomed. J..

[79] Sepahy A.A., Ghazi S., Sepahy M.A. (2011). Cost-effective production and optimization of alkaline xylanase by indigenous *Bacillus mojavensis* AG137 fermented on agricultural waste. Enzym. Res..

[80] Bocchini D.A., Alves-Prado H.F., Baida L.C., Roberto I.C., Gomes E., Da Silva R. (2002). Optimization of xylanase production by *Bacillus circulans* D1 in submerged fermentation using response surface methodology. Process Biochem..

[81] Atalla S.M.M., Ahmed N.E., Awad H.M., El Gamal N.G., El Shamy A.R. (2020). Statistical optimization of xylanase production, using different agricultural wastes by *Aspergillus oryzae* MN894021, as a biological control of faba bean root diseases. Egypt. J. Biol. Pest Control.

[82] Ravindran R., Williams G.A., Jaiswal A.K. (2019). Spent coffee waste as a potential media component for xylanase production and potential application in juice enrichment. Foods.

[83] Geetha K., Gunasekaran P. (2010). Optimization of nutrient medium containing agricultural waste for xylanase production by *Bacillus pumilus* B20. Biotechnol. Bioprocess. Eng..

[84] Long C., Liu J., Gan L., Zeng B., Long M. (2019). Optimization of xylanase production by *Trichoderma orientalis* using corn cobs and wheat bran via statistical strategy. Waste Biomass Valorization.

[85] Senthilkumar S.R., Ashokkumar B., Chandra Raj K., Gunasekaran P. (2005). Optimization of medium composition for alkali-stable xylanase production by *Aspergillus fischeri* Fxn 1 in solid-state fermentation using central composite rotary design. Bioresour. Technol..

[86] Jahnen-Dechent W., Ketteler M. (2012). Magnesium basics. Clin. Kidney J..

[87] Sissi C., Palumbo M. (2009). Effects of magnesium and related divalent metal ions in topoisomerase structure and function. Nucleic Acids Res..

[88] Lee D.-Y.D., Galera-Laporta L., Bialecka-Fornal M., Moon E.C., Shen Z., Briggs S.P., Garcia-Ojalvo J., Süel G.M. (2019). Magnesium flux modulates ribosomes to increase bacterial survival. Cell.

[89] Lusk J.E., Williams R.J.P., Kennedy E.P. (1968). Magnesium and the Growth of *Escherichia coli*. J. Biol. Chem..

[90] Watcharawipas A., Watanabe D., Takagi H. (2018). Sodium acetate responses in *Saccharomyces cerevisiae* and the *Ubiquitin ligase* Rsp5. Front. Microbiol..

[91] Lim Y.H., Foo H.L., Loh T.C., Mohamad R., Abdul Rahim R., Idrus Z. (2019). Optimized medium via statistical approach enhanced threonine production by *Pediococcus pentosaceus* TL-3 isolated from Malaysian food. Microb. Cell Factories.

[92] Kaushal R., Sharma N., Dogra V. (2015). Optimization of the production and molecular characterization of cellulase-free xylanase from an alkalophillic *Bacillus subtilis* SD8 isolated from paper mill effluent. Appl. Biochem. Microbiol..

[93] Patel K., Dudhagara P. (2020). Optimization of xylanase production by *Bacillus tequilensis* strain UD-3 using economical agricultural substrate and its application in rice straw pulp bleaching. Biocatal. Agric. Biotechnol..

[94] Sharma D., Sharma G., Mahajan R. (2019). Development of strategy for simultaneous enhanced production of alkaline xylanase-pectinase enzymes by a bacterial isolate in short submerged fermentation cycle. Enzym. Microb. Technol..

[95] Cubas-Cano E., González-Fernández C., Ballesteros M., Tomás-Pejó E. (2018). Biotechnological advances in lactic acid production by lactic acid bacteria: Lignocellulose as novel substrate. Biofuels Bioprod. Biorefining.

[96] Jiang J., Yang B., Ross R.P., Stanton C., Zhao J., Zhang H., Chen W. (2020). Comparative genomics of *Pediococcus pentosaceus* isolated from different niches reveals genetic diversity in carbohydrate metabolism and immune system. Front. Microbiol..

[97] Raman J., Kim J.-S., Choi K.R., Eun H., Yang D., Ko Y.-J., Kim S.-J. (2022). Application of lactic acid bacteria (LAB) in sustainable agriculture: Advantages and limitations. Int. J. Mol. Sci..

[98] Nwamba M.C., Sun F., Mukasekuru M.R., Song G., Harindintwali J.D., Boyi S.A., Sun H. (2021). Trends and hassles in the microbial production of lactic acid from lignocellulosic biomass. Environ. Technol. Innov..

[99] Carvalho I.P.C.D., Detmann E., Mantovani H.C., Paulino M.F., Valadares Filho S.D.C., Costa V.A.C., Gomes D.I. (2011). Growth and antimicrobial activity of lactic acid bacteria from rumen fluid according to energy or nitrogen source. Rev. Bras. Zootec..

[100] Nel H.A., Bauer R., Vandamme E.J., Dicks L.M. (2001). Growth optimization of *Pediococcus damnosus* NCFB 1832 and the influence of pH and nutrients on the production of pediocin PD-1. J. Appl. Microbiol..

[101] Karne H., Moharir S. (2023). Optimization of lactic acid production from different substrates using *Rhizopus oryzae*. Mater. Today Proc..

[102] Brown D.M., Grunden A.M., Pawlak J.J. (2021). Statistical optimization of black liquor-containing media for growth and lactic acid production by *Paenibacillus glucanolyticus* SLM1. Bioresour. Technol. Rep..

[103] Altaf M., Naveena B.J., Reddy G. (2007). Use of inexpensive nitrogen sources and starch for L(+) lactic acid production in anaerobic submerged fermentation. Bioresour. Technol..

[104] Katepogu H., Wee Y.J., Anu Appaiah K., Chinni S.V., Gopinath S.C.B., Syed A., Verma M., Lebaka V.R. (2023). Lactic acid production by *Pediococcus pentosaceus* HLV1 from banana crop residue: An economic and renewable resource. Biomass Convers. Biorefinery.

[105] Coelho L., De Lima C., Rodovalho C., Bernardo M., Contiero J. (2011). Lactic acid production by new *Lactobacillus plantarum* LMISM6 grown in molasses: Optimization of medium composition. Braz. J. Chem. Eng..

[106] Wang Y., Chen C., Cai D., Wang Z., Qin P., Tan T. (2016). The optimization of L-lactic acid production from sweet sorghum juice by mixed fermentation of *Bacillus coagulans* and *Lactobacillus rhamnosus* under unsterile conditions. Bioresour. Technol..

[107] Heenan C.N., Adams M.C., Hosken R.W., Fleet G.H. (2002). Growth medium for culturing probiotic bacteria for applications in vegetarian food products. LWT—Food Sci. Technol..

[108] Watson S.P., Clements M.O., Foster S.J. (1998). Characterization of the starvation-survival response of *Staphylococcus aureus*. J. Bacteriol..

[109] Cui Y., Qu X. (2021). Genetic mechanisms of prebiotic carbohydrate metabolism in lactic acid bacteria: Emphasis on *Lacticaseibacillus casei* and *Lacticaseibacillus paracasei* as flexible, diverse and outstanding prebiotic carbohydrate starters. Trends Food Sci. Technol..

[110] Pajak B., Siwiak E., Sołtyka M., Priebe A., Zieliński R., Fokt I., Ziemniak M., Jaśkiewicz A., Borowski R., Domoradzki T. (2020). 2-Deoxy-d-Glucose and its analogs: From diagnostic to therapeutic agents. Int. J. Mol. Sci..

[111] Krisnawati R., Cahyanto M.N., Sardjono S., Suroto D.A., Widada J. (2022). RNA-seq data of *Aspergillus tubingensis* NBRC 31125 in carbon catabolite repressor related to xylanase production. Data Brief.

[112] Farliahati M.R., Ramanan R.N., Mohamad R., Puspaningsih N.N.T., Ariff A.B. (2010). Enhanced production of xylanase by recombinant *Escherichia coli* DH5α through optimization of medium composition using response surface methodology. Ann. Microbiol..

[113] Alokika, Singh B. (2020). Enhanced production of bacterial xylanase and its utility in saccharification of sugarcane bagasse. Bioprocess Biosyst. Eng..

[114] Foo H., Loh T., Lai P., Lim Y., Kufli C., Rusul G. (2003). Effects of adding *Lactobacillus plantarum* I-UL4 metabolites in drinking water of rats. Pak. J. Nutr..

[115] Alshelmani M.I., Loh T.C., Foo H.L., Lau W.H., Sazili A.Q. (2014). Biodegradation of palm kernel cake by cellulolytic and hemicellulolytic bacterial cultures through solid state fermentation. Sci. World J..

[116] Nakkarach A., Foo H.L., Song A.A.-L., Nitisinprasert S., Withayagiat U. (2020). Promising discovery of beneficial *Escherichia coli* in the human gut. 3 Biotech.

[117] Borshchevskaya L., Gordeeva T., Kalinina A., Sineokii S. (2016). Spectrophotometric determination of lactic acid. J. Anal. Chem..

[118] Miller G.L. (1959). Use of dinitrosalicylic acid reagent for determination of reducing sugar. Anal. Chem..

